# Early-life undernutrition induces enhancer RNA remodeling in mice liver

**DOI:** 10.1186/s13072-021-00392-w

**Published:** 2021-03-31

**Authors:** Yinyu Wang, Yiting Mao, Yiran Zhao, Xianfu Yi, Guolian Ding, Chuanjin Yu, Jianzhong Sheng, Xinmei Liu, Yicong Meng, Hefeng Huang

**Affiliations:** 1 The International Peace Maternity and Child Health Hospital, School of Medicine, Shanghai Jiao Tong University, Shanghai, China; 2 School of Biomedical Engineering and Technology, Tianjin Medical University, Tianjin, China; 3 Shanghai Key Laboratory of Embryo Original Disease, Shanghai, China; 4 Institute of Embryo-Fetal Original Adult Disease Affiliated To Shanghai, Jiao Tong University School of Medicine, Shanghai, China; 5 Department of Pathology and Pathophysiology, School of Medicine, Zhejiang University, Hangzhou, China

**Keywords:** Early-life undernutrition, Global run-on sequencing, Nascent RNA, Enhancer, Metabolism

## Abstract

**Background:**

Maternal protein restriction diet (PRD) increases the risk of metabolic dysfunction in adulthood, the mechanisms during the early life of offspring are still poorly understood. Apart from genetic factors, epigenetic mechanisms are crucial to offer phenotypic plasticity in response to environmental situations and transmission. Enhancer-associated noncoding RNAs (eRNAs) transcription serves as a robust indicator of enhancer activation, and have potential roles in mediating enhancer functions and gene transcription.

**Results:**

Using global run-on sequencing (GRO-seq) of nascent RNA including eRNA and total RNA sequencing data, we show that early-life undernutrition causes remodeling of enhancer activity in mouse liver. Differentially expressed nascent active genes were enriched in metabolic pathways. Besides, our work detected a large number of high confidence enhancers based on eRNA transcription at the ages of 4 weeks and 7 weeks, respectively. Importantly, except for ~ 1000 remodeling enhancers, the early-life undernutrition induced instability of enhancer activity which decreased in 4 weeks and increased in adulthood. eRNA transcription mainly contributes to the regulation of some important metabolic enzymes, suggesting a link between metabolic dysfunction and enhancer transcriptional control. We discovered a novel eRNA that is positively correlated to the expression of circadian gene *Cry1* with increased binding of epigenetic cofactor p300.

**Conclusions:**

Our study reveals novel insights into mechanisms of metabolic dysfunction. Enhancer activity in early life acts on metabolism-associated genes, leading to the increased susceptibility of metabolic disorders.

**Supplementary Information:**

The online version contains supplementary material available at 10.1186/s13072-021-00392-w.

## Background

The prevalence of metabolic disorders is significantly increasing worldwide. Metabolic syndrome is defined as a combination of central obesity and insulin resistance (IR) with impairment of glucose, blood pressure or lipid metabolism. Both epidemiological [1, 2] and experimental studies [3, 4] have illustrated that adverse environmental influences during the early life of the fetus result in fetal programming, followed by permanent changes in metabolic health in later life [2]. Maternal undernutrition is a key determinant for the delivery of low birth weight (LBW) and transmitting metabolic disorders to the next generation. One fundamental epidemiologic study, presided by David Baker, focused on the inverse association between LBW and high blood pressure in children 10 years old [5, 6]. Based on this association, Barker proposed his theory about the fetal origin of adult disease (FOAD) [7]. In addition, numerous studies [8–10] have demonstrated that LBW increases offspring susceptibility to diabetes and dyslipidemia. A maternal protein restriction diet (PRD) during pregnancy and lactation, an extensively established mouse model of LBW [11], is used to investigate the fetal origin of metabolic disorders.

Although emerging studies [9, 11–13] have shown that maternal PRD increases the risk of metabolic dysfunction in adulthood, the mechanisms during the early life of offspring are still poorly understood. Apart from genetic factors, epigenetic mechanisms are crucial to offer phenotypic plasticity in response to environmental situations and transmission [2, 3, 13]. Transcriptional regulatory elements (TREs), including enhancers and promoters, can determine the transcription levels of associated genes from the beginning. In brief, formation of a preinitiation complex (PIC), including RNA polymerase II (Pol II), is a prerequisite to executing the correct programs of mRNA synthesis [14, 15]. Activated transcription is controlled by activators located in both the promoter-proximal site and distal (enhancer) regulatory site [14], which results in the transcription of enhancer-associated noncoding RNAs (eRNAs) [16]. Only the successful escape of paused Pol II from the promoter-proximal site can lead to elongation during transcription [15, 17]. Based on this physiological process, a series of works [18, 19] confirmed that the promoter-proximal pausing of Pol II is a crucial postrecruitment and rate-limiting step for gene regulation. In addition, eRNA transcription serves as a robust indicator of enhancer activation, and have potential roles in mediating enhancer functions and gene transcription [20–22], which can be applied to understanding the transcriptional regulation of disease, like tumorigenesis [23, 24].

Two studies [25, 26] investigated the epigenetic effects of Pol II in skeletal muscle of maternal PRD female offspring as early as the age of 38 days postnatally. One study found that the increased binding of Pol II at the CCAAT/enhancer-binding protein (C/EBP) promoter region was associated with abnormal energy metabolism. Another study found a significantly increased level of Pol II at the glucose transporter 4 (GLUT4) promoter region, resulting in elevated synthesis of glycogen. These studies [20, 21] suggest that TREs, including enhancers, may play pivotal roles in the development of metabolic disease.

Global run-on sequencing (GRO-seq) can determine whether Pol II is merely promoter-bound or engaged in transcription [17, 27]. Another major advantage of GRO-seq is the achievement of measuring enhancer activity based on eRNA transcription through its generally low stability and abundance [21]. In this study, GRO-seq was used to map and quantify transcriptionally engaged Pol II density across the genome in vitro. Combined with RNA-seq, we characterized the global alterations of eRNAs and their active genes, revealed enhancers involved in regulating the fetal origin of metabolic dysfunction.

## Results

### Animal model construction and liver global run-on sequencing library preparation

**Fig. 1:**
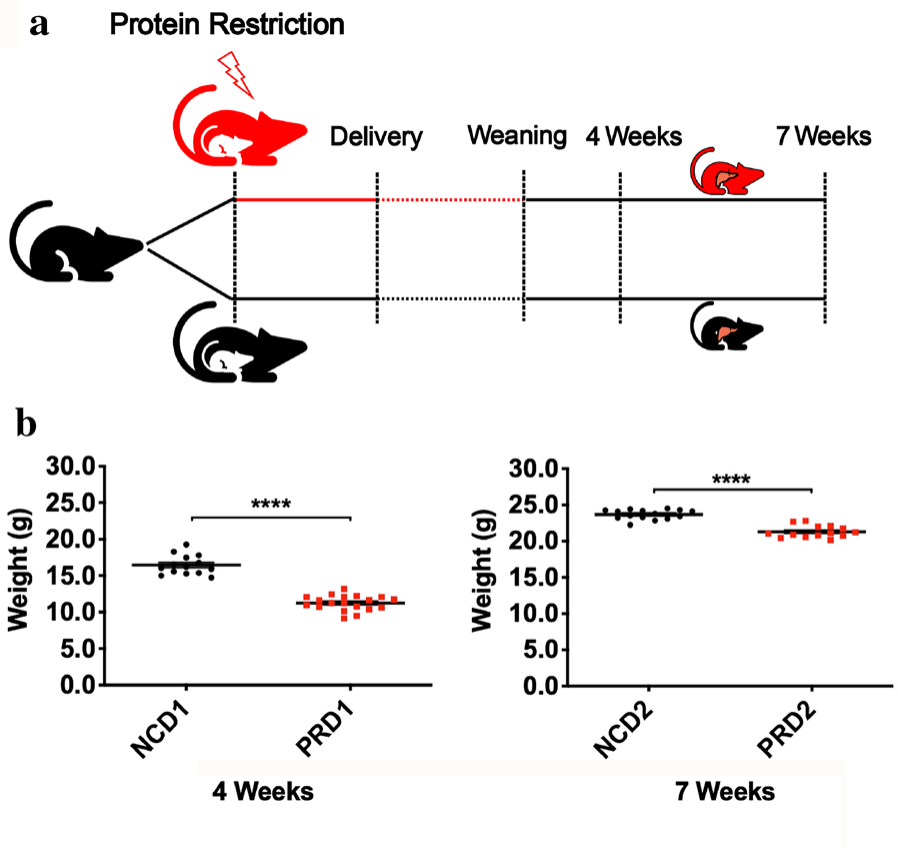
Overview of animal model. **a** The schema of animal model construction. **b** Comparison of body weight between NCD and PRD male offspring at the ages of 4 weeks (NCD1 vs PRD1) and 7 weeks (NCD2 vs PRD2), respectively. All values are presented as mean ± SEM. *p < 0.05, ****p < 0.0001 (Student’s *t* test)

First, we established a PRD animal model as shown in Fig. 1a. Briefly, pregnant female mice were randomly divided into two groups once the vaginal plugs were detected. One group was fed normal chow diet (NCD), and the other group was fed a PRD from pregnancy to the end of lactation. At day 21, both male pups were weaned from the mother to normal chow. Pup body weight was measured, and liver samples were pooled together from four mice each at the age of 4 weeks (NCD1 and PRD1) and 7 weeks (NCD2 and PRD2) for later library preparation. All the body weights of the male offspring born to PRD-fed mice were significantly lower than those of the controls, as shown in Fig. 1b. Moreover, the body weight gap narrowed from 4 to 7 weeks, which suggested that catch-up growth happened among PRD male offspring. To clarify the effect of maternal PRD on offspring livers, we optimized a procedure employing a global run-on sequencing strategy to detect changes in nascent RNA transcription. Specifically, mouse livers were first cut into pieces and then homogenized with a glass homogenizer in different buffers [28]. After a series of centrifugations, the liver nuclei were ultimately isolated. The characteristic morphology of nuclei was retained after isolation (see Additional file 1: Figure S1A and S1C). cDNA template ranging from 150 to 500 base pairs (bp) were extracted (Additional file 1: Figure S2). Amplified cDNA products ranging from 200 to 500 bp were extracted from TBE gel for further assays, as illustrated in Additional file 1: Figure S1B and S1D. Using the Illumina high-throughput sequencing platform, we obtained approximately 45–68 million reads that can be aligned to the repeat-unmasked genome (Additional file 2: Table S1).

### Early-life undernutrition alters hepatic nascent RNA transcription

**Fig. 2:**
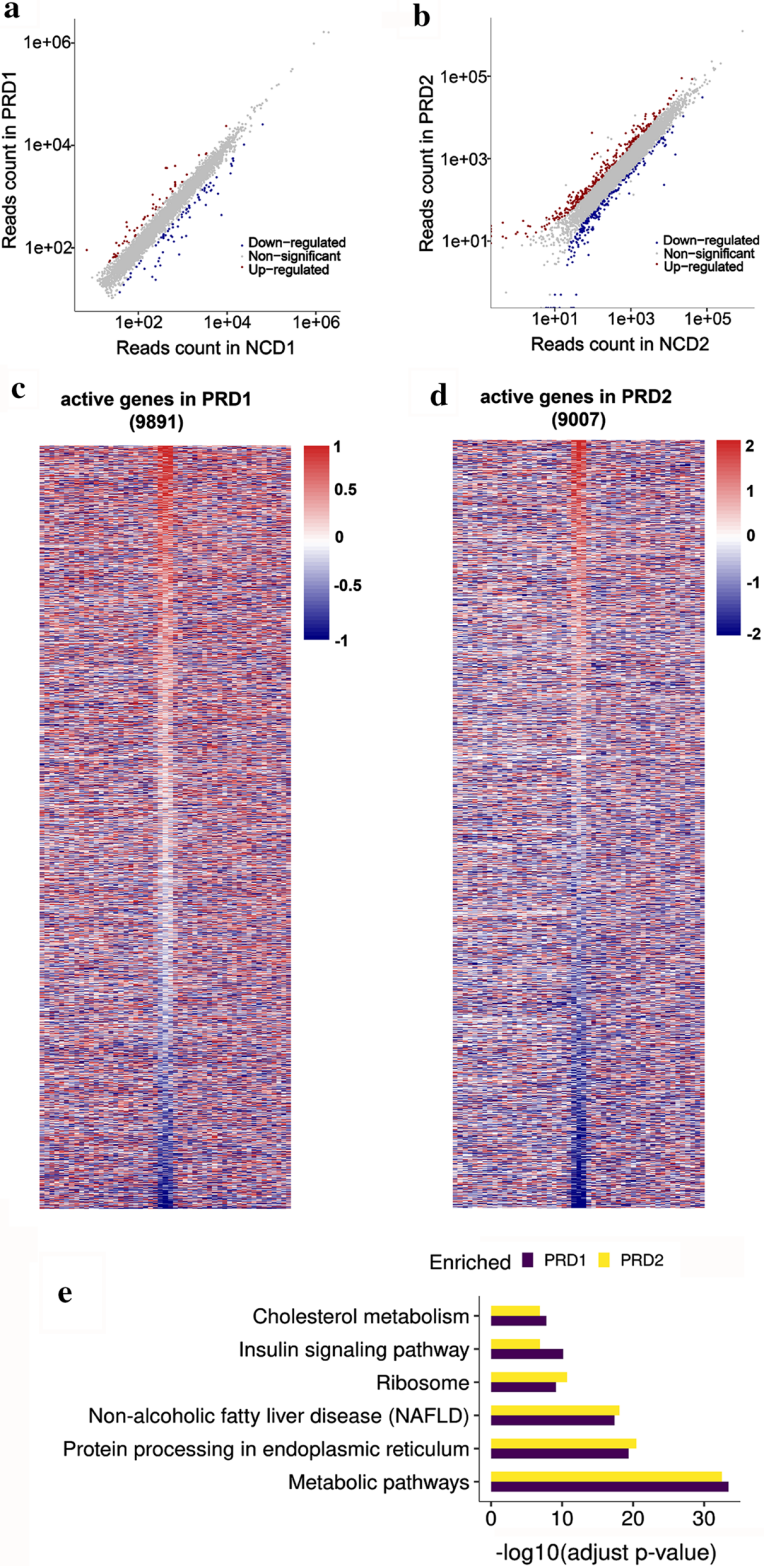
Impact of maternal PRD on nascent transcripts. **a**, **b** The dot plot graph shows all differentially expressed nascent RNA based on the fold change and *p* value. Blue dots represent down-regulated transcription and red dots represent up-regulated transcription. **c**, **d** Heatmap of log_2_-transformed fold changes of RNA polymerases ± 5 kb from TSSs with 200 bp bin size for all nascent active genes from maternal PRD and NCD livers at the ages of 4 weeks and 7 weeks, respectively. **e** Differentially expressed genes between the offspring of the dams fed the PRD and NCD at the age of 4 weeks and 7 weeks in GRO-seq were analyzed by Kyoto Encyclopedia of Genes and Genomes (KEGG). Fold change > 1.5, *p* < 0.05

A general profiling of nascent transcription was then obtained from the GRO-seq data at 4 weeks and 7 weeks of PRD livers. Before the reads mapping, the adapter ligation product and ployA were removed and only reads that above 32 bp were kept (Additional file 2: Table S1). Then, the clean reads were mapped to mouse genome (mm10, see Methods). Gene expression levels represented by normalized read counts were used to measure differential gene expression. The differentially expressed transcription increased from 4 to 7 weeks of age, as reflected by comparing the amounts of colored dots, as shown in Fig. 2a, b. Transcriptionally active genes were determined using the criteria of promoter-proximal (pp) density greater than zero and gene body (gb) density greater than 4 reads/kb after total read count is normalized to 10 Mb based on the background estimation [22]. Therefore, 9891 active genes from 4 weeks and 9007 active genes from 7 weeks of male offspring liver nuclei induced by PRD were identified, as shown in Fig. 2c, d and Additional file 3: Table S2. By the use of the Kyoto Encyclopedia of Genes and Genomes (KEGG) database, the PRD-specific active genes were enriched in several metabolic pathways, inducing those associated with protein processing in the endoplasmic reticulum, nonalcoholic fatty liver disease (NAFLD), ribosomes, insulin signaling and cholesterol metabolism, in the livers of both 4-week-old and 7-week-old mouse, as shown in Fig. 2e. Most of these pathways correlated with nutrient metabolism, which demonstrated that PRD male offspring were at high risk for later metabolic abnormalities. Interestingly, KEGG analysis for RNA-seq data of PRD1 and PRD2 offspring liver showed no consistency in enriched pathways (Additional file 1: Figure S3). These data suggest that nascent RNA change mainly contributes to the high risk of metabolism disorder.

Furthermore, to analyze all active genes at multiple stages, including transcription initiation, elongation and pausing, we calculated the transcriptional signals at pp and gb which stand for the position of Pol II. Pol II usually pauses at the promoter-proximal site before the release of Pol II into productive transcription. Moreover, it is generally accepted that promoter-proximal pausing of Pol II is one of the indicators of transcriptional activity. Therefore, the alterations in Pol II density were identified for 807 genes in 7-week-old PRD offspring (Additional file 1: Figure S4A). Nevertheless, the expression of the majority of these genes did not change despite the altered Pol II density of their corresponding promoter-proximal regions. Besides, the PRD-induced promoter-proximal changed genes were enriched in ribosome, metabolic pathways, protein processing in endoplasmic reticulum and glycine, serine and threonine metabolism in the livers of 7-week-old mouse (Additional file 1: Figure S4B).

### Identification of enhancers in the abundant nascent RNAs

**Fig. 3:**
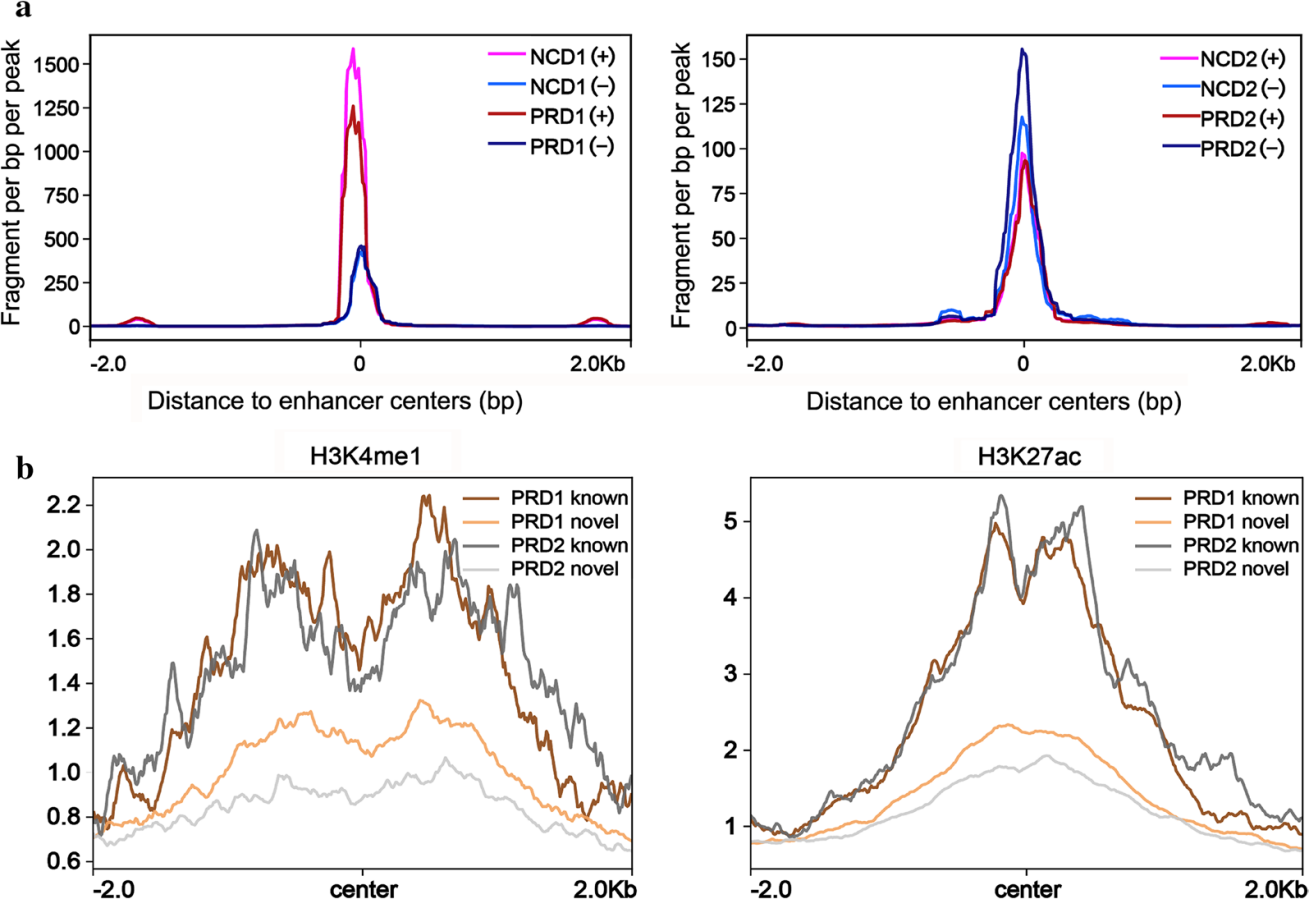
Active enhancers identified in the mouse liver. **a** Signals for identified enhancers on plus and minus strands, respectively. **b** Enrichment of H3K4me1 and H3K27ac for known and novel enhancers by the use of FANTOM5

A major advantage of GRO-seq is the simultaneous determination of enhancer activity according to eRNA transcription. The density of read peaks mapped uniquely around the center of enhancers on both plus and minus strands, as shown in Fig. 3a. After preliminary identification (see Methods), 7184 unannotated eRNAs and 350 annotated eRNAs (4595 eRNAs in PRD1 and 4599 eRNAs in PRD2, including 1660 eRNAs in common) (see Additional file 3: Table S2) were obtained. To minimize the noise and focus on the biological meanings, the significant changed enhancers were filtered by |log_2_(Fold Change)|> 1 and adjusted *p* value < 0.05. In total, 1921 and 1275 enhancers shared were obtained from two independent replicates prepared from 4-week-old and 7-week-old mouse livers, respectively (Additional file 1: Figure S5), suggesting high quality of library construction and high reproducibility of enhancer identification. Furthermore, de novo eRNA loci are typically enriched for other epigenomic features of histone modification [17, 21, 28], such as monomethylation of histone H3 lysine 4 (H3K4me1), p300 [29] and acetylation of histone H3 lysine 27 (H3K27ac). The former two represent enhancer identity, and the latter indicates activity. Only enhancers that can be linked to the candidate target genes were kept for display and annotation. Novel enhancers were defined if their centers do not fall in any enhancers region based on the FANTOM5 database. Highly matched signal peaks of public H3K4me1 and H3K27ac data from mouse liver with our GRO-seq data are illustrated in Fig. 3b. Finally, apart from 350 annotated eRNAs, our work also revealed 7184 high confidence unannotated eRNAs by overlapping with either active enhancer mark, H3K4me1 and H3K27ac (Fig. 3b).

### Enhancer activity in early life acts on metabolism-associated genes

**Fig. 4:**
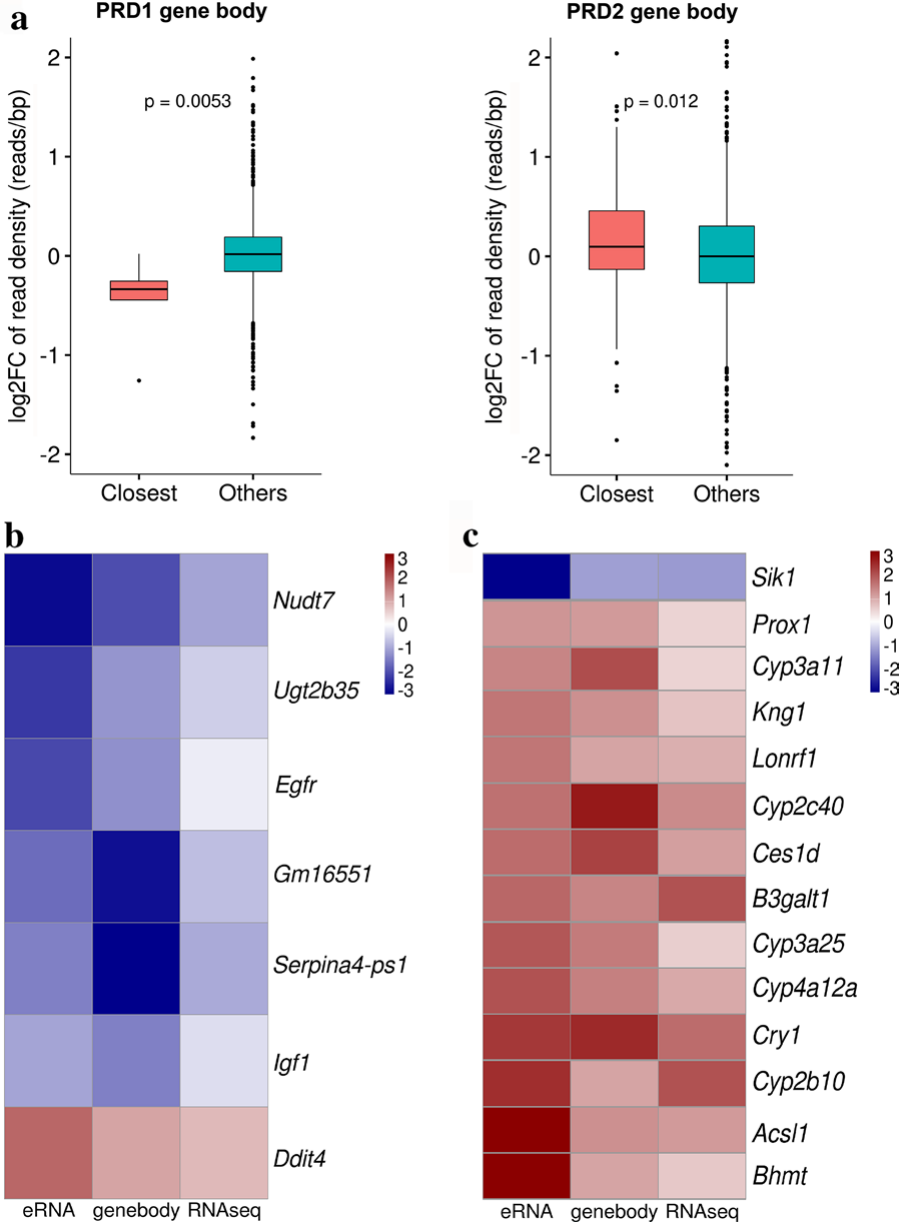
Enhancer remodeling regulates metabolic gene activity. **a** Correlation of RNA transcriptional abundance in gene body regions associated with down- (PRD1) and up- (PRD2) regulated enhancers for the closest and other active genes. **b**, **c** Genes whose genebody |log_2_(Fold Change)|> 1 and adjusted *p* < 0.05 were filtered as candidates for heatmap of their eRNA and RNA-seq data generated by IMAGE based on their differential transcription in PRD1 (**b**) and PRD2 (**c**)

The presence of eRNAs correlates with enhancer activity. eRNAs are unstable for steady-state accumulation, by performing GRO-seq, we identified ~ 1000 remodeling enhancers in PRD male mouse offspring (Additional file 3: Table S2 and S3). Enhancers are key transcriptional regulators to enable spatial control of gene expression, and the active genes closest to enhancers showed much higher transcriptional activity in both pp and gb regions than other active genes [22]. Therefore, we analyzed the distribution of RNA transcription abundance in gb of enhancer-associated genes (closest) and other active genes in PRD1 and PRD2. These results showed that the down-regulated enhancer in PRD1 is significantly related to down regulation in the gb regions for the closest active genes (*p* = 0.0053), and the up-regulated enhancer in PRD2 showed significantly higher transcriptional activity in the gb regions for the closest active genes (*p* = 0.012) (Fig. 4a). But there was no significant correlation between either up-regulated enhancers in PRD1 or down-regulated enhancers in PRD2 with their gb regions for the closest active genes (*p* = 0.15 in PRD1, *p* = 0.85 in PRD2) (see Additional file 1: Figure S6). This result suggests that differential expression of nearest genes in GRO-seq are regulated by their enhancers.

Based on genebody expression between NCD and PRD (select standard: log_2_(Fold Change)|> 1), their corresponding eRNAs and RNA-seq heatmap results were generated by IMAGE in PRD1 and PRD2 (weighted *p* value < 0.05). Enhancer activity was down-regulated in 4 weeks, then up-regulated in 7 weeks, consistent with the eRNA signal peak value around enhancer center in four samples (Fig. 3a). Those significant changed genes (gb log_2_(Fold Change)|> 1) showed high consistency between eRNA, genebody and RNA-seq expression (Figs. 4b and 4c). Basically, most of these genes are metabolic enzymes that related to lipid metabolism and other metabolic process (see Table 1). Serum analysis was performed for 7-week-old mice (PRD2), HDL was lower in PRD offspring (Additional file 1: Figure S7).

**Table 1:** Early-life undernutrition induced nascent transcription alteration of metabolic enzymes

Metabolic enzymes	*Cyp3a11*	Cytochrome P450 3A11
	*Cyp2c40*	Cytochrome P450 2C40
	*Cyp3a25*	Cytochrome P450 3A25
	*Cyp4a12a*	Cytochrome P450 4A12A
	*Cyp2b10*	Cytochrome P450 2B10
	*Sik1*	Salt Inducible Kinase 1
	*Nudt7*	Nudix Hydrolase 7
	*Ugt2b35*	UDP-glucuronosyltransferase
	*Bhmt*	Betaine--Homocysteine S-Methyltransferase
	*B3galt1*	Beta-1,3-Galactosyltransferase 1
	*Acsl1*	Acyl-CoA Synthetase Long Chain Family Member 1
	*Ces1d*	Carboxylesterase 1D

Significantly changed genes related to metabolic pathways were selected to compare their expression between GRO-seq and RNA-seq, both showed high consistency between eRNA, genebody and RNA-seq expression at the same ages PRD mice

For all differential expressed eRNAs (without selection by log_2_(Fold Change)|> 1 for genebody), Q-PCR was performed to validate the expression of their corresponding genebody. Ten randomly selected genes were detected in liver total RNA, the RNA levels (Additional file 1: Figure S8A) showed the same trend with their eRNAs levels (Additional file 1: Figure S8B).

We then mined eRNAs to uncover transcription factors (TFs) responsible for transcription activity influenced by PRD. However, only nuclear receptor subfamily 5 group A member 2 (NR5A2) motif was enriched at the sites of down-regulated eRNAs in livers of PRD1 mouse (Additional file 4: Table S4). Besides, there are 19 motifs were enriched at the sites of up-regulated eRNAs in liver of PRD2 (Additional file 4: Table S4). Interestingly, the down-regulated eRNAs in liver of PRD1 were most enriched for NR5A2 motif. The orphan nuclear receptor NR5A2 is involved in a wide variety of biologic processes, including cholesterol and glucose metabolism in the liver [30], such as regulating the expression of sterol 12α-hydrolyase (CYP8B1) and cholesterol ester transport protein (CETP). These results suggested that the upstream TRE activity regulation in early stage might increase the susceptibility of metabolic disorders among PRD male offspring. Enhancer mediates metabolic gene transcription in early-life undernutrition.

*Nudt7* and *Serpina4-ps1*, each of which was randomly selected from the 10 above mentioned genes (Additional file 1: Figure S8), were down-regulated due to their eRNAs down regulation as early as 4 weeks of age, as shown in Fig. 4b. Because eRNAs are often unstable and traditional transcriptome profiling such as RNA-seq cannot capture these transcripts [21], therefore, we compare the results of GRO-seq with RNA-seq. The gene body transcriptional changes were consistent with our RNA-seq result (see Additional file 1: Figure S9). According to the results of the RNA-seq assay, these two genes were located on chromosomes 8 and 12, respectively. The GRO-seq assay results of the two groups were compared with the RNA-seq assay results, apparently low signals were detected at the corresponding sites in the PRD group. When previously published H3K27ac data derived from mouse liver were used, the down-regulated novel enhancers were characterized at the same regions. The decreased signals of GRO-seq at the enhancer region, illustrated by H3K27ac peaks, and corresponding downregulation were observed in the chromosome region in the mouse liver RNA-seq data, indicating the alterations of the enhancer correlated to the transcriptional change. Our study reveals novel insights into mechanisms of early-life undernutrition induced metabolic dysfunction. Enhancer activity in early life acts on metabolism-associated genes, leading to the increased susceptibility of metabolic disorders among PRD male offspring.

### A novel eRNA regulates the expression of core circadian gene *Cry1*

**Fig. 5:**
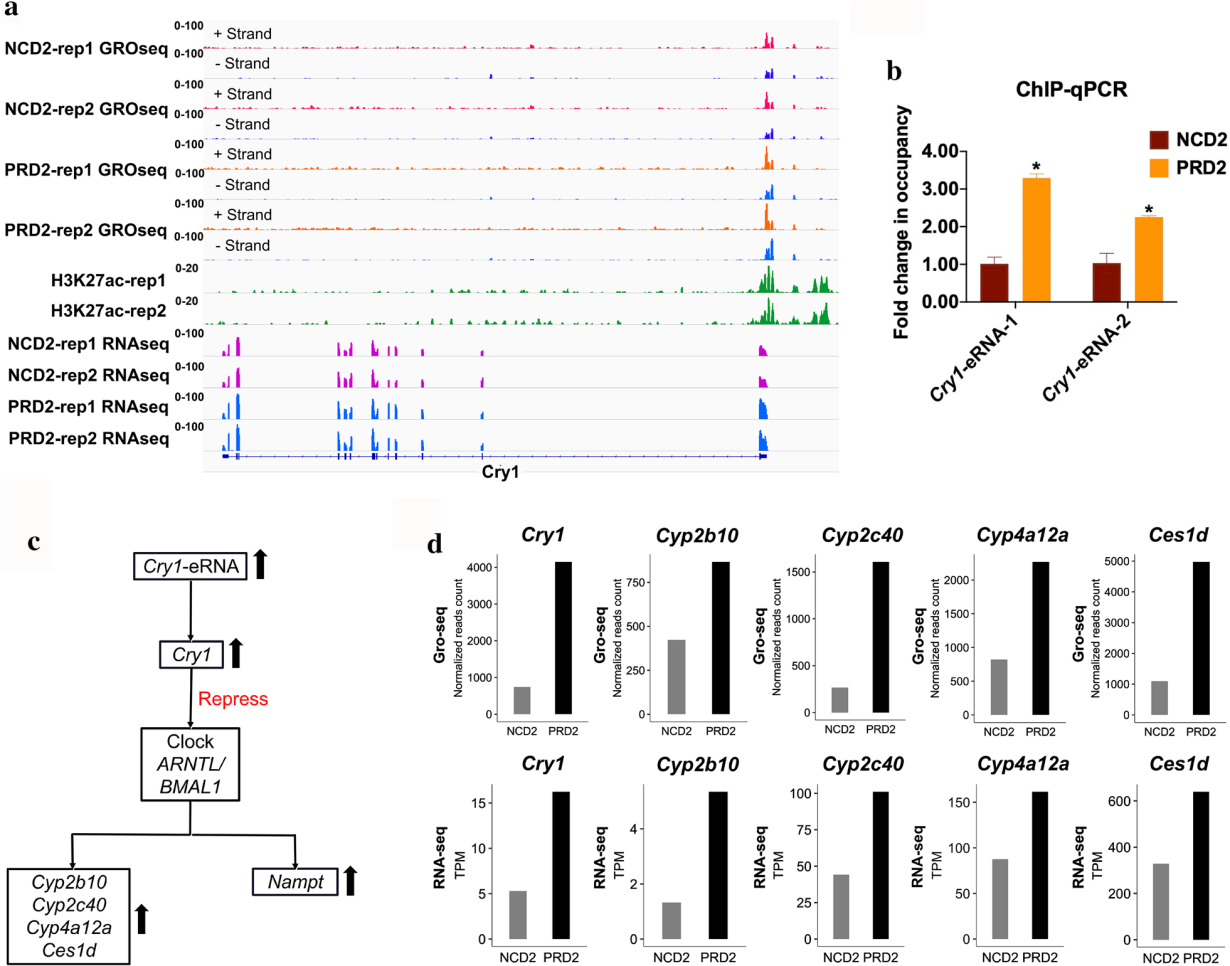
A novel enhancer RNA (eRNA) regulates the expression of core circadian gene *Cry1. a* IGV snapshot of sequencing data, identified novel enhancer on plus and minus strands, H3K27ac peaks, and RNA-seq data of *Cry1*. This novel enhancer (chr10: 85198257–85199349) regulates *Cry1* expression directly. **b** Chromatin immunoprecipitation (ChIP)-Q-PCR assays for recruitment to *Cry1* eRNA transcription site in NCD2 and PRD2 using ideal p300 antibody. *Cry1*-eRNA-1 and *Cry1*-eRNA-2 are two different primers for *Cry*-eRNA transcription site on the chromatin. **c** A key gene regulatory relationship between *Cry1* and its downstream genes. Black arrow shows up-regulated RNA level detected in this study. **d** The high-throughput GRO-seq and RNA-seq data were calculated in column chart

This study reveals significantly changed nascent transcription of metabolism enzyme genes in the liver of PRD offspring (Fig. 4b, c, and Table 1). Furthermore, we discovered a novel eRNA regulating core circadian gene *Cry1* expression in 7-week-old mouse liver (Fig. 4c and Additional file 1: Figure S8). To illustrate the results clearly, we also supplied information for strand specific nascent RNAs in *Cry1* (Fig. 5a and Additional file 5: Table S5). Even though the transcription sites for this novel eRNA were enriched by H3K27ac peaks from other published data in mouse liver, this eRNA was not detected before (Fig. 5a). eRNA can activate gene transcription in multicellular organisms. For instance, transcription of enhancer RNAs affect the expression of cognate genes through transcriptional interference or modulation of chromatin structure in promoter regions [31]. To further detect whether this up-regulated novel eRNA is accompanied by increasing enhancer regulatory components in PRD2, ChIP-Q-PCR was performed for p300 and p300 was dramatically increased in the eRNA transcription site on the chromatin (Fig. 5b).

As one of the core circadian clock component, CRY1 plays a critical role in rhythm generation. CRY1 are transcriptional repressors by forming the negative limb of the feedback loop and interact with the CLOCK-ARNTL/BMAL1 [32–35]. In accordance with current study [36], repressing the CLOCK-ARNTL/BMAL1 induced significant transcription of NAMPT. NAMPT was indeed significantly up-regulated in PRD offspring (Additional file 1: Figure S8). Another study showed that Clock ablation disrupted hepatic diurnal expressions of a number of drug-metabolizing enzymes and regulated drug detoxification [37]. In particular, Clock ablation sensitized mice to cyclophosphamide toxicity by upregulating CYP2B10-mediated metabolism (generating the toxic metabolite 4-hydroxycyclophosphamide) [37]. Based on current studies *Cry1* regulates *Cyp2b10*, *Cyp2c40*, *Cyp4a12a*, *Ces1d* and *Nampt* by repressing Clock ARNTL/BMAL1 (Fig. 5c). Common to these findings is the correlation between the levels of RNA-seq and GRO-seq for *Cyp2b10*, *Cyp2c40*, *Cyp4a12a* and *Ces1d* (Fig. 5d). Enhancer activity in early life acts on metabolism-associated genes, leading to the increased susceptibility of metabolic disorders.

## Discussion

Although many studies have shown that metabolic disorders are amplified in old individuals, fetal programming occurs at a relatively early age. PRD can contribute increased glucose levels to male offspring in intraperitoneal glucose tolerance tests as early as weaning age [8, 9]. In addition, another mouse study [10] showed glucose intolerance and impaired insulin secretion in 8-week-old male offspring. One rat model study [38] showed that the hepatic glycogen content was markedly increased in PRD offspring at 26 days, while glycogen synthase expression had already been elevated 16 days before. Besides, previous studies have demonstrated that male offspring are prone to be influenced by PRD in young adulthood [2, 13, 39]. However, the mechanisms of metabolic risk during the early life of PRD offspring are still poorly understood, so we collected the liver tissues of 4-week-old and 7-week-old male offspring to study metabolic-related transcriptional changes during the early life period.

Liver is the most critical tissue responsible for nutrient intake and energy demands in humans and rodents. In the present study, we found ∼9000 active genes—4595 and 4599 differentially expressed enhancers in PRD offspring mice at 4 and 7 weeks, respectively (Fig. 2c, d and Additional file 3: Table S2). In fact, our study indicates that enhancers down-regulated in response to PRD in male pups at 4 weeks contribute to the decreased expression of their nearest genes (Fig. 4b). The closest active genes displayed significantly decreased transcriptional activity in gene body regions caused by maternal PRD during pregnant (Fig. 4a). Importantly, our study demonstrated general down-regulated eRNA of metabolic-associated genes in the livers of 4-week-old PRD male offspring, contributing to significantly decreased expression of their nearest genes. A thrifty hypothesis [2, 4, 38] postulates that these metabolic adaptations occur to ensure a sufficient supply of energy to vital organs, such as brain development. However, the expression of most of these genes returned to normal at the age of 7 weeks, or they were even overexpressed, indicating that these changes may be beneficial for starvation but detrimental during times of sufficient nutrition later in life. But the total enhancer activity was up-regulated at 7 weeks caused by catch-up growth (Fig. 3a and b). The body weight gap between PRD offspring and the control was reduced from 4 to 7 weeks of age, which also suggests that the PRD offspring grew faster than the control offspring in our animal model. Numerous studies [2, 3, 11] have supported catch-up growth as being an important risk factor accelerating the development of metabolic syndrome. In our study, the fetal origin of metabolic dysfunction model induces the instability of enhancers, and their relative genes are mainly enriched in metabolic pathways (Fig. 2e). This new observation might be one of the possible mechanisms by which male offspring born to PRD are at high risk of developing metabolic disorders.

In this study, we illustrated the potential involvement of eRNA in affecting the expression of two metabolism-associated genes, *Gm16551* and *Serpina4-ps1*, as a consequence of PRD (Fig. 4b). One study [40] confirmed that *Gm16551* is negatively regulated by sterol regulatory element-binding protein 1C (SREBP-1C), which is associated with lipogenesis in mouse liver. SREBP-1C is a master transcription factor of lipogenic genes and is known to be involved in a wide range of cellular processes of mTORC1 signaling, such as proliferation, metabolism, inflammation and aging. In our case [41], we previously found enhanced levels of mTORC1 signaling upon PRD in male offspring splenic CD4^+^ T cells. Additionally, offspring born to PRD manifested defects in mTOR cell signaling both in human and rodent studies [2, 42]. *Serpina4-ps1* is a pseudogene of the serine proteinase inhibitor superfamily that originates from clade A member 4 [43]. The functional gene *Serpina4*, also known as kallistatin, is abundant in the liver and a potent vasodilatory protein involved in the prevention of cardiovascular disease [44]. Recently, a case–control study [45] demonstrated that *Serpina4* was down-regulated in the liver of obese NAFLD patients and plays critical roles in the inhibition of inflammation and oxidative stress.

Additional work is needed to determine the effects of PRD on metabolic nascent RNA in offspring within a specific time window. Both gestation and lactation are critical periods during early life programming of the fetus; however, we did not investigate which stage is more important in influencing eRNA profiles. As previously described in our laboratory [41], the consumption of prenatal protein restriction may exhibit more profound effects than postnatal protein restriction. Additionally, another study [3] illustrated that consuming a PRD during gestation results in IR in both the first and second generation, whereas consuming a PRD during lactation influences only the second generation.

An intriguing finding in our work is the 807 genes with changes in the promoter-proximal region discovered in 7-week-old PRD male offspring livers (Additional file 1: Figure S4). Generally, the accumulation of Pol II is supposed to be a sign of transcriptional activity. However, similar to those of many studies [15, 17], our results revealed that the majority of Pol II pausing was simply due to transcriptional engagement but prevented elongation, resulting in unchanged expression of their corresponding gene body regions. On one hand, the density of Pol II in promoter-proximal regions is determined by the balance between entry and escape rates [19]. The higher density of paused Pol II is simply reflected by the entry rate being faster than the escape rate. However, transcripts in gene body regions accumulated much more slowly than those in promoter-proximal regions, leading to independent transcriptional change between those two regions [22]. On the other hand, the pausing of Pol II at promoter-proximal regions may have other functions beyond transcriptional initiation. One possible reason is that paused Pol II is a feature of open promoters and could significantly accelerate the expression of genes in response to environmental change [18]. Another reason is Pol II pauses to prepare for efficient activation with other coactivators [21, 27].

The markedly high number of novel eRNAs that were identified in this work is an unexpected observation. One reason for this unusual discovery is that we focused on metabolic risk during the early life of PRD male offspring; this phenomenon has not been studied in such a way before (for its nascent RNA transcriptional regulation). It is thus likely that there are other novel eRNAs are associated with other models and remain to be identified.

## Conclusions

The results presented here constitute the first comprehensive dataset of liver nascent RNA transcriptional regulation in PRD offspring. These novel findings shed light on the mechanism in susceptibility of the fetal origin of metabolic disorders induced by enhancers.

## Methods

### Animal model and tissue collection

The animal experiment was performed as previously described [41]. In brief, all mice (C57BL/6J) used in this work were purchased from SLAC Laboratory Animal Company (Shanghai, China). All animals were bred at room temperature at 22 ± 2 °C under a 12-h light/dark cycle, with free access to food and water. Virgin female C57BL/6J mice were mated with male mice. The following morning, pregnant dams were randomly assigned to either a provision of standard normal chow diet (NCD; 20% protein) or a protein restriction diet (8% protein; FBSH Diets Services, Shanghai, China). The female mice were maintained on these respective diets until their offspring were weaned onto a standard diet. Upon weaning, all of the litters were adjusted to 4–5 pups each to ensure that no litter was nutritionally biased. Only male offspring mice in both groups were sacrificed by cervical dislocation after CO_2_ narcosis at 4 weeks and 7 weeks of age. The liver tissues were immediately quickly removed, frozen in liquid nitrogen and stored at − 80 °C for later use. All the experiments were approved by the Institutional Animal Care and Use Committee of Shanghai Jiao Tong University School of Medicine (Approval ID: B-2016-025).

### Serum analysis

Blood collection was performed via canthal vein puncture. Serum was obtained after clotting and centrifugation (5000*g*, 15 min). Serum samples were sent to the Service bio technology company for analysis. The following serum assays were completed using Rayto chemray-240 to the manufacturer’s instructions: free fatty acids, triglycerides, total cholesterol, HDL-cholesterol, LDL-cholesterol and nonesterified fatty acid (NEFA).

### Preparation of the global run-on sequencing (GRO-seq) library and sequencing

GRO-seq was performed as previously described [28]. Briefly, 600 mg of liver tissues from four mice were subjected to dounce in cold swelling buffer. Single nuclei were made with lysis buffer and resuspended in freezing buffer. For the run-on assay, nuclei were mixed with nuclear run-on buffer (NRO) to extend nascent RNAs. To specifically isolate RNA, we added 5-bromouridine-5′-triphosphate (BrUTP) (Biotium, San Francisco, USA) to BrU-tagged nascent RNA during the run-on step, and nuclear RNA was extracted with TRIzol LS (Life Technologies Corp.). Then, the NRO RNA was chemically hydrolyzed into short fragments (∼100 bases) and purified through a p-30 column (BIO-RAD, California, USA). BrU-containing NRO RNA was selected through immunopurification with anti-BrU agarose beads (Santa Cruz Biotechnology, California, USA). These RNAs were modified via polyadenylation, and reverse transcription was performed to generate cDNA. NRO cDNA was circularized and then relinearized, ssDNA template was amplified by PCR, and the product was run on a nondenaturing polyacrylamide TBE gel (Life Technologies Corp.). The library was then subjected to Illumina paired-end 2 × 150 sequencing. The primers used in the library are summarized in Additional file 6: Table S6.

### RNA sequencing (RNA-seq)

RNA-seq was performed on the livers of PRD mice at the age of 4 weeks and 7 weeks. Total RNA was extracted using TRIzol (Life Technologies Corp.) and further treated with DNase to remove genomic DNA contamination. Isolation of mRNA was performed using a NEB Next PolyA mRNA Magnetic Isolation Module (New England Biolabs, Ipswich, MA, USA), and the mRNA was then used for RNA-seq library preparation with a NEB Next Ultra Directional RNA Library Prep Kit for Illumina (New England Biolabs, Ipswich, MA, USA). The library was then subjected to Illumina sequencing with paired-end 2 × 150 as the sequencing mode.

### Q-PCR

Total RNA was extracted from 10 to 20 mg liver tissues with the FastPure Cell/Tissue Total RNA Isolation Kit V2 (Vazyme, Nanjing, China), and cDNA was prepared using the PrimeScript RT reagent Kit with gDNA Eraser (Takara Bio, Shiga, Japan). Quantitative PCR was performed with TB Green Premix Ex Taq (Tli RNaseH Plus; Takara Bio) on a Quant Studio 7 Flex (Life Technologies, Grand Island, NY), and primers for *Lpin1*, *Ppargc1a*, *Sulf2*, *Nampt*, *B3galt*, *Dio3os*, *Cry1*, *Nudt7*, *Serpina-ps1*, and *Acsl1* were from Primer Bank (see Additional file 7: Table S7). Data were normalized to *Actin*, and results are shown as a fold induction relative to expression levels in age-matched normal chow animals, as indicated.

### ChIP-Q-PCR

Cells were crosslinked with 1% formaldehyde for 10 min at room temperature and quenched with 125 mM glycine. The fragmented chromatin fragments were pre-cleared and then immunoprecipitated with Protein A + G Magnetic beads coupled with anti-p300 antibodies (Cell Signaling Technology, MA, USA, #54062). After reverse crosslinking, ChIP and input DNA fragments were used directly for qPCR with *Cry1* (10:85185100–85185350, 10:85185150–85185300). Primers for the Q-PCR were used to amplify the *Cry1* enhancer sequences (Additional file 7: Table S7).

### Data analysis

To analyze GRO-seq Nascent transcripts, the Illumina sequencing reads were mapped using BWA (0.7.17-r1188) onto the repeat-masked mouse reference genome mm10, allowing a maximum of two mismatches in the reads. Only uniquely mapped reads were considered for further analysis. NRSA (v2, http://bioinfo.vanderbilt.edu/NRSA/) was used to analyze nascent transcription profiles generated by GRO-seq data [22]. For the GRO-seq data between PRD and NCD at the age of 4 weeks and 7 weeks separately, DESeq2 was used to identify the differentially expressed genes as the RNA-seq. All sequencing libraries were carried out with pooled liver samples that from four randomly selected mouse, and each analysis consisted of two technical replicates. Gene expression levels represented by normalized reads count were used to measure differential gene expression. The false discovery rate (FDR) control method was used to calculate the adjusted *p* value in multiple testing to evaluate the significance of the differences. Here, only gene with an adjusted *p* value < 0.05 were used for subsequent analysis.

Active genes were identified using the criteria of promoter-proximal density greater than zero and gene body density greater than 4 reads/kb [22]. The changes of RNA polymerases ± 5 kb from TSSs with 200 bp bin size for all active genes comparing PRD and NCD mouse livers was visualized using heatmap plot, while genes were ranked by promoter-proximal density changes. Promoter-proximal changed genes were calculated by the same criterion.

Enhancer identification was finished using previously published analysis method [22]. Briefly, enhancers were detected and quantified based on novel transcripts called by HOMER. Before enhancer calling, the intergenic transcripts within regions − 2 kb to + 20 kb from any RefSeq gene were excluded, because regions within − 2 kb from transcription start sites (TSSs) are generally considered as promoter regions, and RNA polymerases transcribe beyond annotated transcription termination sites (TTSs). Then, enhancers were annotated based on the FANTOM5 database. The enhancers were linked to candidate target genes using several strategies, and the newest strategy was used in our analysis. Finally, the expression changes of enhancers across conditions were estimated using DESeq2. The details in each step can be found in the NRSA paper. To minimize the noise and focus on the biological meanings, only the significant changed enhancers in the Venn diagram were used for the following analysis. The significant changed enhancers were filtered by |log2(Fold Change)|> 1 and adjusted *p*-value < 0.05. Besides, only enhancers which can be linked to the candidate target genes were kept for the display and annotation. Wilcoxon signed-rank test was used for analyzing the distribution of transcriptional changes in promoter-proximal and gene-body regions for the closest active genes associated with up-regulated enhancers and other active genes. Profiles were compared to detect whether identified enhancers were marked by enhancer-associated histone modification signatures (H3K4me1 and H3K27ac). H3K4me1 and H3K27ac ChIP-seq data in the mouse liver were obtained from ENCODE (H3K4me1: ENCSR000CAO, H3K27ac: ENCSR000CDH). Pathway enrichment analysis is a functional analysis that maps genes to Kyoto Encyclopedia of Genes and Genomes (KEGG) pathways. The adjusted *p* value denotes the significance of the pathway correlated to the conditions.

For RNA-seq data, raw reads were filtered to obtain high-quality clean reads by removing sequencing adapters, short reads (length < 35 bp) and low-quality reads adopt Cutadapt (v2.10). FastQC is used to ensure high reads quality. The clean reads were mapped to the mouse genome (assembly GRCm38) using the HISAT2^[4]^ software. Then, the DESeq2 was used to do the differential gene expression analysis based on the negative binomial distribution. Genes whose |log_2_(Fold Change)|> 1 and adjusted *p* value < 0.05 were filtered as differentially expressed genes (DEGs).

To identify TFs enriched in the loci of PRD-specific eRNAs, eRNAs within 200 kb of PRD1 genes were used for motif analysis using HOMER [46]. The eRNAs peaks were classified into three categories based on the expression change of eRNAs, up-regulated, down-regulated, and non-significant. The peaks of non-significant eRNAs were used as the background. The motif analysis was focused on peaks of up-regulated eRNAs. The motifs were found using the exact sizes of peaks with the repeat-masked mouse genome sequence (mm10) by findMotifsGenome.pl from HMOER. Only motifs that could be mapped to mouse transcription factors were included, and redundant motifs were removed. Motifs were clustered, merged, and trimmed using methods as previously described [47].

## Supplementary Information

**Additional file 1: Figures S1–S10.** Figure S1. Overview of GRO-seq library construction. A, C Isolated liver nuclei stained with DAPI. B, D Amplified DNA library range from 200–500 bp. Figure S2. Agarose gel extraction for cDNA fragment from 150nt-500nt in GRO-seq library construction. Figure S3. Impact of maternal PRD on total transcripts. Differentially expressed genes between the offspring of the dams fed the PRD and NCD at the age of 4 weeks (A) and 7 weeks (B) in RNA-seq were analyzed by Kyoto Encyclopedia of Genes and Genomes (KEGG). Fold Change  > 1.5, *p* < 0.05. Figure S4. Promoter-proximal changed genes in PRD2. A Heatmap of log_2_-transformed fold changes in RNA polymerases ± 5 kb from TSSs with 200 bp bin size for genes showing significant change in RNA polymerases in promoter-proximal regions (pp up: upregulated in promoter-proximal regions; pp. down: downregulated in promoter-proximal regions; gb up: upregulated in gene body region; gb down: downregulated in gene body region; gb unchanged: unchanged in gene body region). B Promoter-proximal changed genes between the offspring of the dams fed the PRD and NCD at the age of 7 weeks were analyzed by Kyoto Encyclopedia of Genes and Genomes (KEGG). fold change > 1.5, *p* < 0.05. Figure S5. High confidence enhancers identification. Overlap of enhancers identified in the mouse liver GRO-seq from two independent replicates prepared from 4 and 7 weeks, respectively. Figure S6. Correlation of RNA transcriptional abundance in gene body regions associated with up- (PRD1) and down- (PRD2) regulated enhancers for the closest and other active genes. Figure S7. Examination of serum lipids profiles for 7-week-old PRD mice. Figure S8. Validation results for the change of enhancers-induced metabolic genes. A Q-PCR detection for 10 randomly selected genes in total RNA of NCD1 vs PRD1, and NCD2 vs PRD2 mice livers. B Heatmap of 10 randomly chosen eRNA expression results generated by IMAGE based on their transcription in PRD1 and PRD2 (weighted *p* < 0.05). Figure S9. Visualization of enhancer induced metabolic gene transcription in PRD. IGV snapshot of sequencing data, identified novel enhancer, H3K27ac peaks, and RNA-seq data of *Nudt7*, *Serpina4-ps1* and *Cyp2b10*. These novel enhancers (chr8: 114091772–114095641; chr12: 104066247–104069323; chr7: 25890844–25894913) contribute to altered expression of their closest genes in PRD. Figure S10. Correlation between different replications of GRO-seq (A) and RNA-seq (B) libraries, respectively.

**Additional file 2: Tables S1.** The number of raw and clean reads obtained from GRO-seq data.

**Additional file 3: Table S2**–**S3.** Table S2. Details of the nascent transcription from GRO-seq data. Table S3. Differentially expressed (up and down regulation) eRNAs of 4 weeks (PRD1) and 7 weeks (PRD2), respectively

**Additional file 4: Table S4.** Motif enrichment at sites of down/up-regulated eRNAs in livers.

**Additional file 5: Table S5.** Peak signals for *Cry1* eRNA on both strands.

**Additional file 6: Table S6.** Details of the library primers.

**Additional file 7: Table S7.** Sequences for real-time PCR primers in this study.

**Additional file 8: Table S8–S9.** Characteristics of all enhancers respect to strands from GRO-seq of PRD1 (Table S8) and PRD2 (Table S9).

## Data Availability

The datasets and computer code produced in this study are available in the following databases: RNA-Seq data: Sequence Read Archive PRJNA664278 (https://www.ncbi.nlm.nih.gov/sra/PRJNA664278). GRO-Seq data: Sequence Read Archive PRJNA664280 (https://www.ncbi.nlm.nih.gov/sra/PRJNA664280).

## Abbreviations

NCD: Normal chow diet
PRD: Protein restriction diet
LBW: Low birth weight
FOAD: Fetal origin of adult disease
TRE: Transcriptional regulatory element
PIC: Preinitiation complex
Pol II: RNA Polymerase II
eRNA: Enhancer-associated noncoding RNA
pp: Promoter-proximal
gb: Gene body
bp: Base pair
TF: Transcription factor
C/EBP: CCAAT/enhancer-binding protein
GLUT4: Glucose transporter 4
GRO-seq: Global run-on sequencing
KEGG: Kyoto Encyclopedia of Genes and Genomes
NAFLD: Non-alcoholic fatty liver disease
IR: Insulin resistance
NR5A2: Nuclear receptor subfamily 5 group A member 2
CYP8B1: Sterol 12α-hydrolyase
CETP: Cholesterol ester transport protein
SREBP1c: Sterol regulatory element-binding protein 1c
FDR: False discovery rate
