# Supplementary figures and images for: Early-life undernutrition induces enhancer RNA remodeling in mice liver
Source: Epigenetics Chromatin. 2021 Mar 31;14:18. doi: 10.1186/s13072-021-00392-w (PMC8011416; doi:10.1186/s13072-021-00392-w)

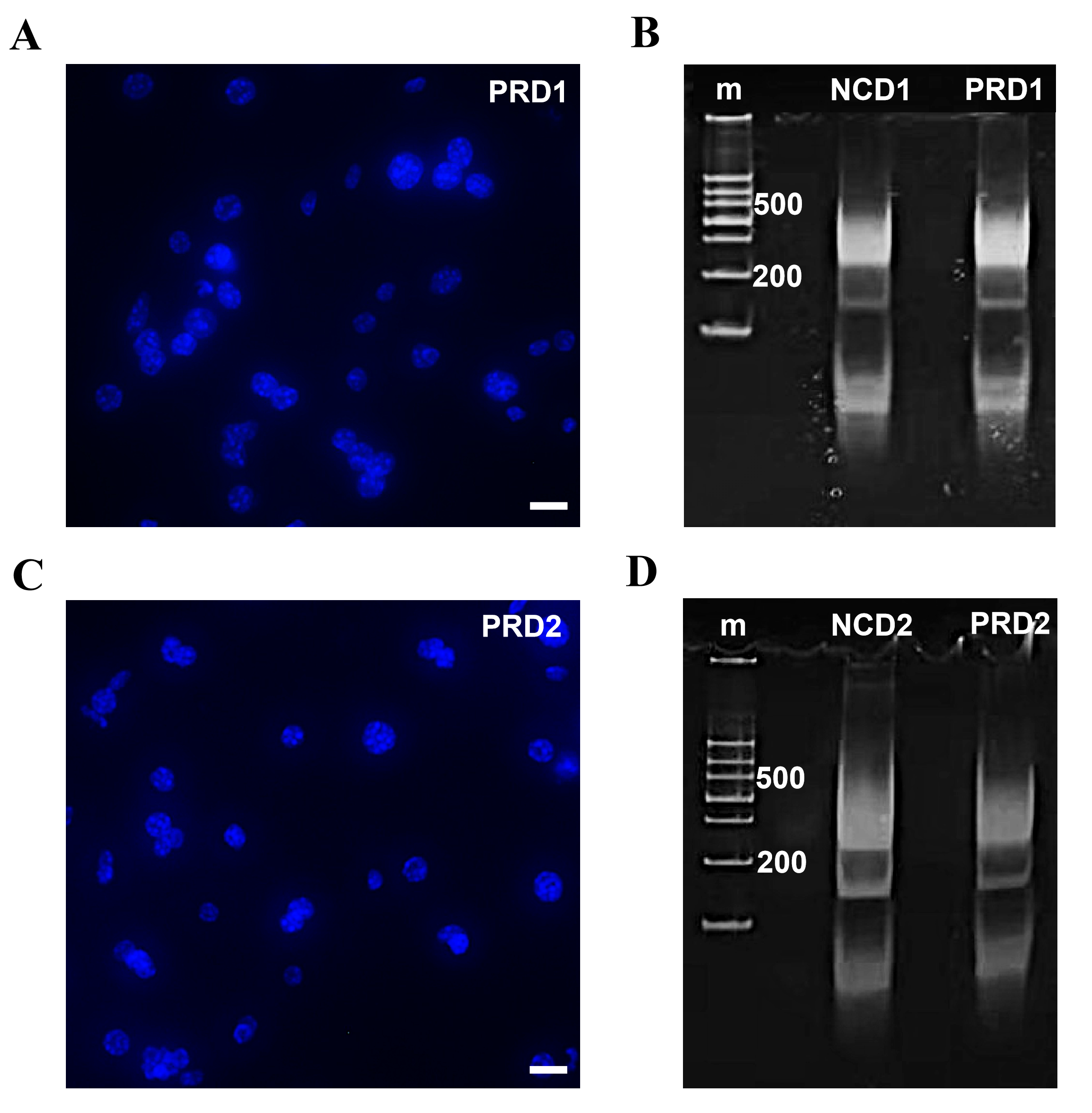

Supplement: Supplementary file 1 — Additional file 1: Figures S1–S10. Figure S1. Overview of GRO-seq library construction. A, C Isolated liver nuclei stained with DAPI. B, D Amplified DNA library range from 200–500 bp. Figure S2. Agarose gel extraction for cDNA fragment from 150nt-500nt in GRO-seq library construction. Figure S3. Impact of maternal PRD on total transcripts. Differentially expressed genes between the offspring of the dams fed the PRD and NCD at the age of 4 weeks (A) and 7 weeks (B) in RNA-seq were analyzed by Kyoto Encyclopedia of Genes and Genomes (KEGG). Fold Change > 1.5, p < 0.05. Figure S4. Promoter-proximal changed genes in PRD2. A Heatmap of log2-transformed fold changes in RNA polymerases ± 5 kb from TSSs with 200 bp bin size for genes showing significant change in RNA polymerases in promoter-proximal regions (pp up: upregulated in promoter-proximal regions; pp. down: downregulated in promoter-proximal regions; gb up: upregulated in gene body region; gb down: downregulated in gene body region; gb unchanged: unchanged in gene body region). B Promoter-proximal changed genes between the offspring of the dams fed the PRD and NCD at the age of 7 weeks were analyzed by Kyoto Encyclopedia of Genes and Genomes (KEGG). fold change > 1.5, p < 0.05. Figure S5. High confidence enhancers identification. Overlap of enhancers identified in the mouse liver GRO-seq from two independent replicates prepared from 4 and 7 weeks, respectively. Figure S6. Correlation of RNA transcriptional abundance in gene body regions associated with up- (PRD1) and down- (PRD2) regulated enhancers for the closest and other active genes. Figure S7. Examination of serum lipids profiles for 7-week-old PRD mice. Figure S8. Validation results for the change of enhancers-induced metabolic genes. A Q-PCR detection for 10 randomly selected genes in total RNA of NCD1 vs PRD1, and NCD2 vs PRD2 mice livers. B Heatmap of 10 randomly chosen eRNA expression results generated by IMAGE based on their transcription in PRD1 and PR [file 13072_2021_392_MOESM1_ESM.zip › Figure S1.tif]

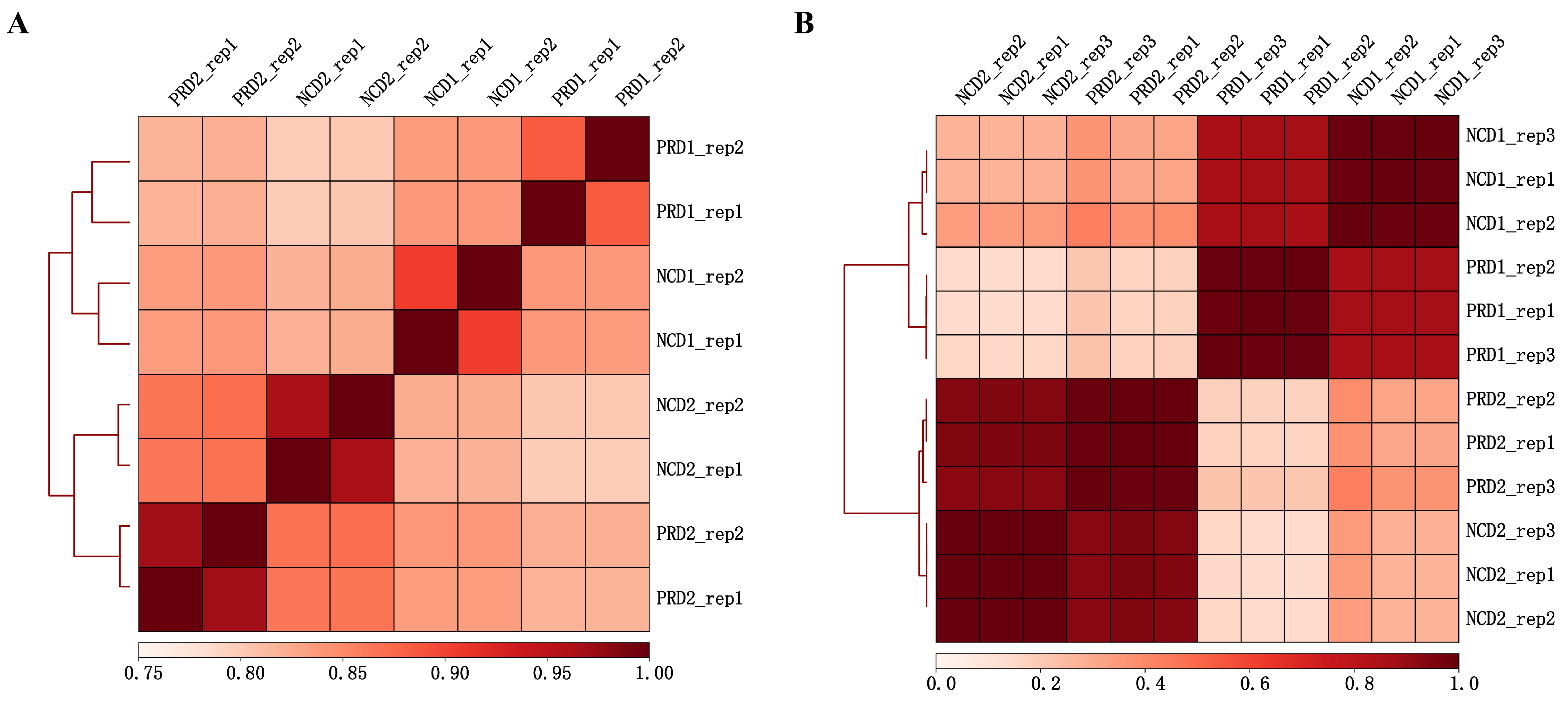

Supplement: Supplementary file 1 — Additional file 1: Figures S1–S10. Figure S1. Overview of GRO-seq library construction. A, C Isolated liver nuclei stained with DAPI. B, D Amplified DNA library range from 200–500 bp. Figure S2. Agarose gel extraction for cDNA fragment from 150nt-500nt in GRO-seq library construction. Figure S3. Impact of maternal PRD on total transcripts. Differentially expressed genes between the offspring of the dams fed the PRD and NCD at the age of 4 weeks (A) and 7 weeks (B) in RNA-seq were analyzed by Kyoto Encyclopedia of Genes and Genomes (KEGG). Fold Change > 1.5, p < 0.05. Figure S4. Promoter-proximal changed genes in PRD2. A Heatmap of log2-transformed fold changes in RNA polymerases ± 5 kb from TSSs with 200 bp bin size for genes showing significant change in RNA polymerases in promoter-proximal regions (pp up: upregulated in promoter-proximal regions; pp. down: downregulated in promoter-proximal regions; gb up: upregulated in gene body region; gb down: downregulated in gene body region; gb unchanged: unchanged in gene body region). B Promoter-proximal changed genes between the offspring of the dams fed the PRD and NCD at the age of 7 weeks were analyzed by Kyoto Encyclopedia of Genes and Genomes (KEGG). fold change > 1.5, p < 0.05. Figure S5. High confidence enhancers identification. Overlap of enhancers identified in the mouse liver GRO-seq from two independent replicates prepared from 4 and 7 weeks, respectively. Figure S6. Correlation of RNA transcriptional abundance in gene body regions associated with up- (PRD1) and down- (PRD2) regulated enhancers for the closest and other active genes. Figure S7. Examination of serum lipids profiles for 7-week-old PRD mice. Figure S8. Validation results for the change of enhancers-induced metabolic genes. A Q-PCR detection for 10 randomly selected genes in total RNA of NCD1 vs PRD1, and NCD2 vs PRD2 mice livers. B Heatmap of 10 randomly chosen eRNA expression results generated by IMAGE based on their transcription in PRD1 and PR [file 13072_2021_392_MOESM1_ESM.zip › Figure S10.tif]

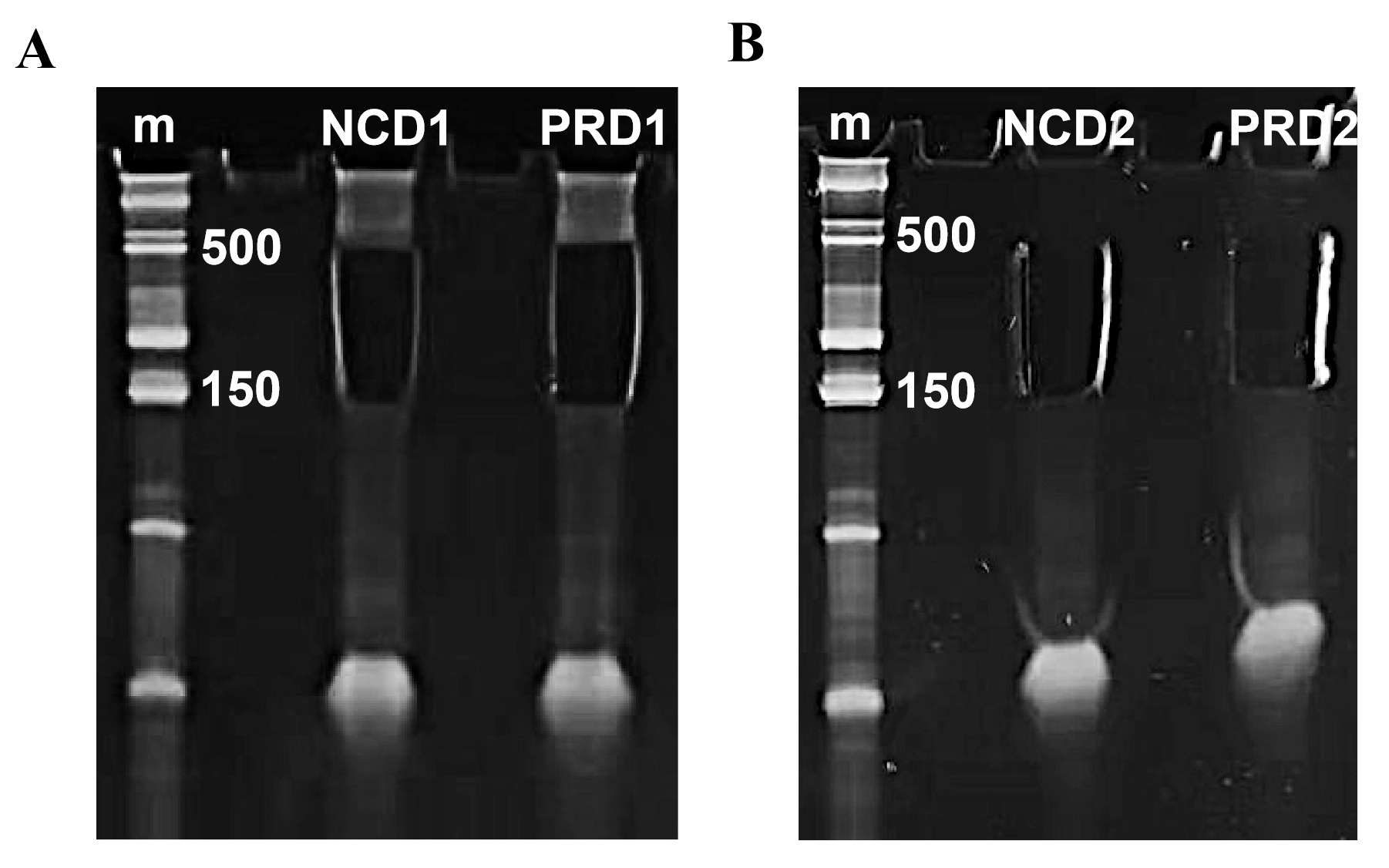

Supplement: Supplementary file 1 — Additional file 1: Figures S1–S10. Figure S1. Overview of GRO-seq library construction. A, C Isolated liver nuclei stained with DAPI. B, D Amplified DNA library range from 200–500 bp. Figure S2. Agarose gel extraction for cDNA fragment from 150nt-500nt in GRO-seq library construction. Figure S3. Impact of maternal PRD on total transcripts. Differentially expressed genes between the offspring of the dams fed the PRD and NCD at the age of 4 weeks (A) and 7 weeks (B) in RNA-seq were analyzed by Kyoto Encyclopedia of Genes and Genomes (KEGG). Fold Change > 1.5, p < 0.05. Figure S4. Promoter-proximal changed genes in PRD2. A Heatmap of log2-transformed fold changes in RNA polymerases ± 5 kb from TSSs with 200 bp bin size for genes showing significant change in RNA polymerases in promoter-proximal regions (pp up: upregulated in promoter-proximal regions; pp. down: downregulated in promoter-proximal regions; gb up: upregulated in gene body region; gb down: downregulated in gene body region; gb unchanged: unchanged in gene body region). B Promoter-proximal changed genes between the offspring of the dams fed the PRD and NCD at the age of 7 weeks were analyzed by Kyoto Encyclopedia of Genes and Genomes (KEGG). fold change > 1.5, p < 0.05. Figure S5. High confidence enhancers identification. Overlap of enhancers identified in the mouse liver GRO-seq from two independent replicates prepared from 4 and 7 weeks, respectively. Figure S6. Correlation of RNA transcriptional abundance in gene body regions associated with up- (PRD1) and down- (PRD2) regulated enhancers for the closest and other active genes. Figure S7. Examination of serum lipids profiles for 7-week-old PRD mice. Figure S8. Validation results for the change of enhancers-induced metabolic genes. A Q-PCR detection for 10 randomly selected genes in total RNA of NCD1 vs PRD1, and NCD2 vs PRD2 mice livers. B Heatmap of 10 randomly chosen eRNA expression results generated by IMAGE based on their transcription in PRD1 and PR [file 13072_2021_392_MOESM1_ESM.zip › Figure S2.tif]

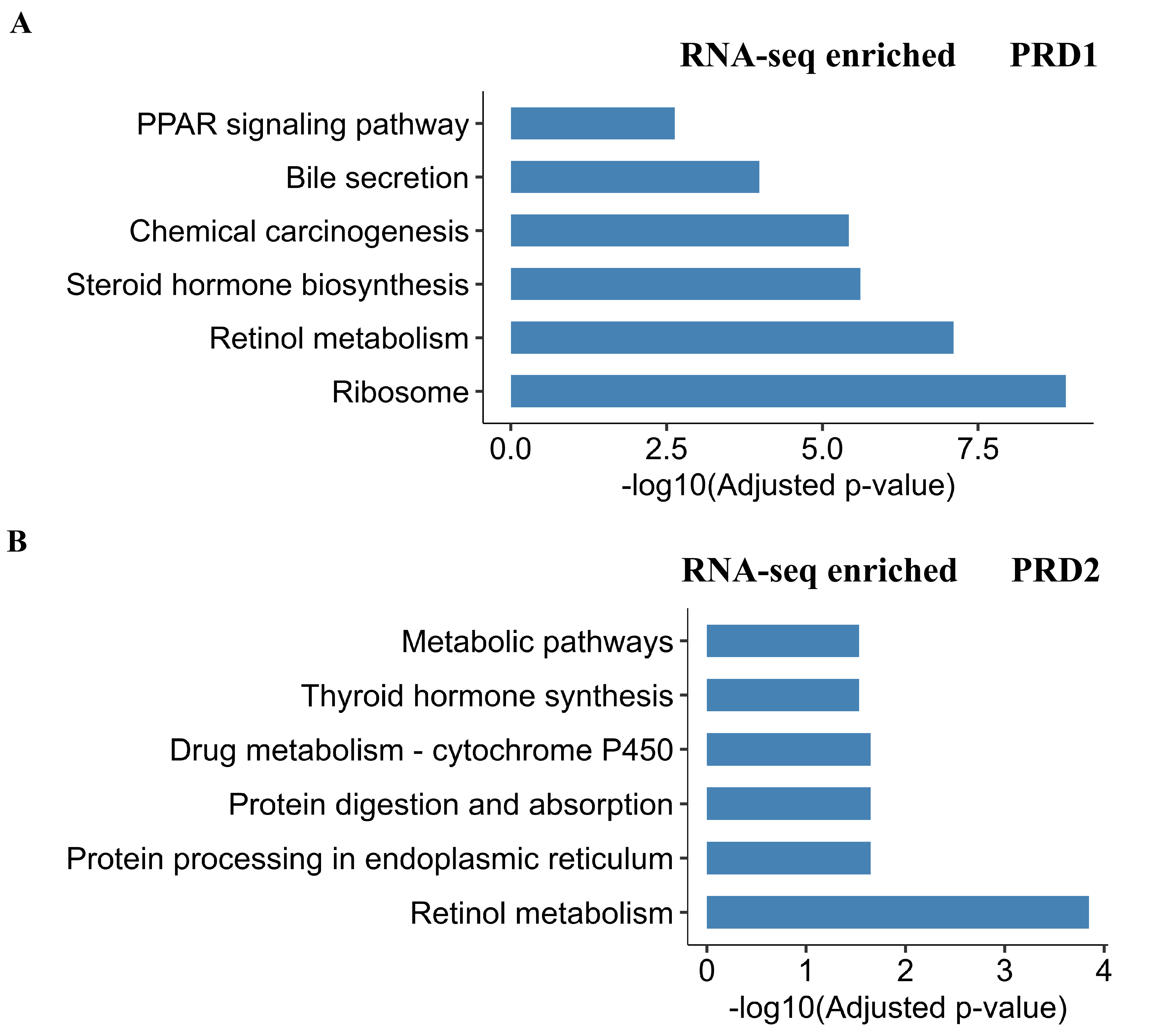

Supplement: Supplementary file 1 — Additional file 1: Figures S1–S10. Figure S1. Overview of GRO-seq library construction. A, C Isolated liver nuclei stained with DAPI. B, D Amplified DNA library range from 200–500 bp. Figure S2. Agarose gel extraction for cDNA fragment from 150nt-500nt in GRO-seq library construction. Figure S3. Impact of maternal PRD on total transcripts. Differentially expressed genes between the offspring of the dams fed the PRD and NCD at the age of 4 weeks (A) and 7 weeks (B) in RNA-seq were analyzed by Kyoto Encyclopedia of Genes and Genomes (KEGG). Fold Change > 1.5, p < 0.05. Figure S4. Promoter-proximal changed genes in PRD2. A Heatmap of log2-transformed fold changes in RNA polymerases ± 5 kb from TSSs with 200 bp bin size for genes showing significant change in RNA polymerases in promoter-proximal regions (pp up: upregulated in promoter-proximal regions; pp. down: downregulated in promoter-proximal regions; gb up: upregulated in gene body region; gb down: downregulated in gene body region; gb unchanged: unchanged in gene body region). B Promoter-proximal changed genes between the offspring of the dams fed the PRD and NCD at the age of 7 weeks were analyzed by Kyoto Encyclopedia of Genes and Genomes (KEGG). fold change > 1.5, p < 0.05. Figure S5. High confidence enhancers identification. Overlap of enhancers identified in the mouse liver GRO-seq from two independent replicates prepared from 4 and 7 weeks, respectively. Figure S6. Correlation of RNA transcriptional abundance in gene body regions associated with up- (PRD1) and down- (PRD2) regulated enhancers for the closest and other active genes. Figure S7. Examination of serum lipids profiles for 7-week-old PRD mice. Figure S8. Validation results for the change of enhancers-induced metabolic genes. A Q-PCR detection for 10 randomly selected genes in total RNA of NCD1 vs PRD1, and NCD2 vs PRD2 mice livers. B Heatmap of 10 randomly chosen eRNA expression results generated by IMAGE based on their transcription in PRD1 and PR [file 13072_2021_392_MOESM1_ESM.zip › Figure S3.tif]

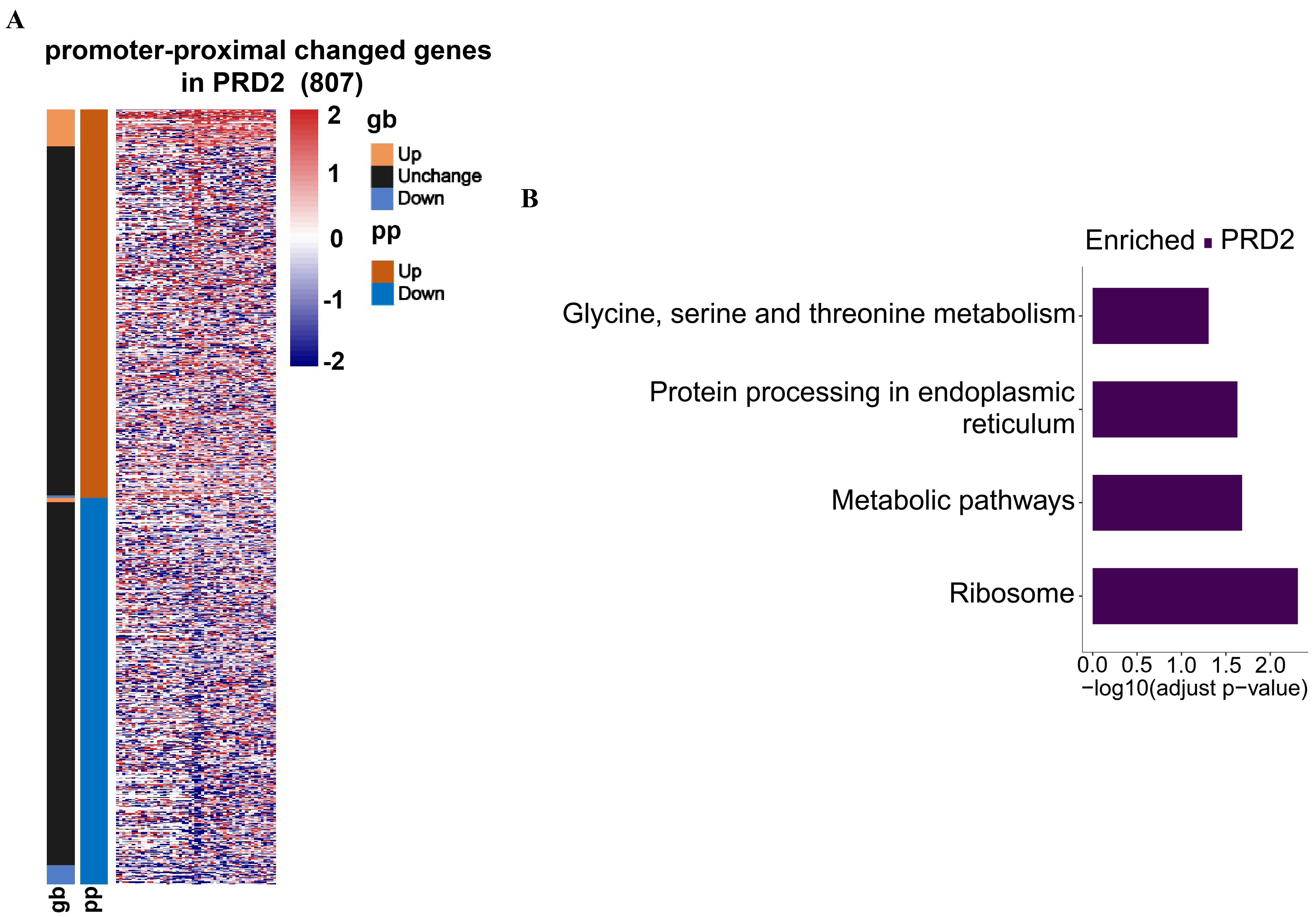

Supplement: Supplementary file 1 — Additional file 1: Figures S1–S10. Figure S1. Overview of GRO-seq library construction. A, C Isolated liver nuclei stained with DAPI. B, D Amplified DNA library range from 200–500 bp. Figure S2. Agarose gel extraction for cDNA fragment from 150nt-500nt in GRO-seq library construction. Figure S3. Impact of maternal PRD on total transcripts. Differentially expressed genes between the offspring of the dams fed the PRD and NCD at the age of 4 weeks (A) and 7 weeks (B) in RNA-seq were analyzed by Kyoto Encyclopedia of Genes and Genomes (KEGG). Fold Change > 1.5, p < 0.05. Figure S4. Promoter-proximal changed genes in PRD2. A Heatmap of log2-transformed fold changes in RNA polymerases ± 5 kb from TSSs with 200 bp bin size for genes showing significant change in RNA polymerases in promoter-proximal regions (pp up: upregulated in promoter-proximal regions; pp. down: downregulated in promoter-proximal regions; gb up: upregulated in gene body region; gb down: downregulated in gene body region; gb unchanged: unchanged in gene body region). B Promoter-proximal changed genes between the offspring of the dams fed the PRD and NCD at the age of 7 weeks were analyzed by Kyoto Encyclopedia of Genes and Genomes (KEGG). fold change > 1.5, p < 0.05. Figure S5. High confidence enhancers identification. Overlap of enhancers identified in the mouse liver GRO-seq from two independent replicates prepared from 4 and 7 weeks, respectively. Figure S6. Correlation of RNA transcriptional abundance in gene body regions associated with up- (PRD1) and down- (PRD2) regulated enhancers for the closest and other active genes. Figure S7. Examination of serum lipids profiles for 7-week-old PRD mice. Figure S8. Validation results for the change of enhancers-induced metabolic genes. A Q-PCR detection for 10 randomly selected genes in total RNA of NCD1 vs PRD1, and NCD2 vs PRD2 mice livers. B Heatmap of 10 randomly chosen eRNA expression results generated by IMAGE based on their transcription in PRD1 and PR [file 13072_2021_392_MOESM1_ESM.zip › Figure S4.tif]

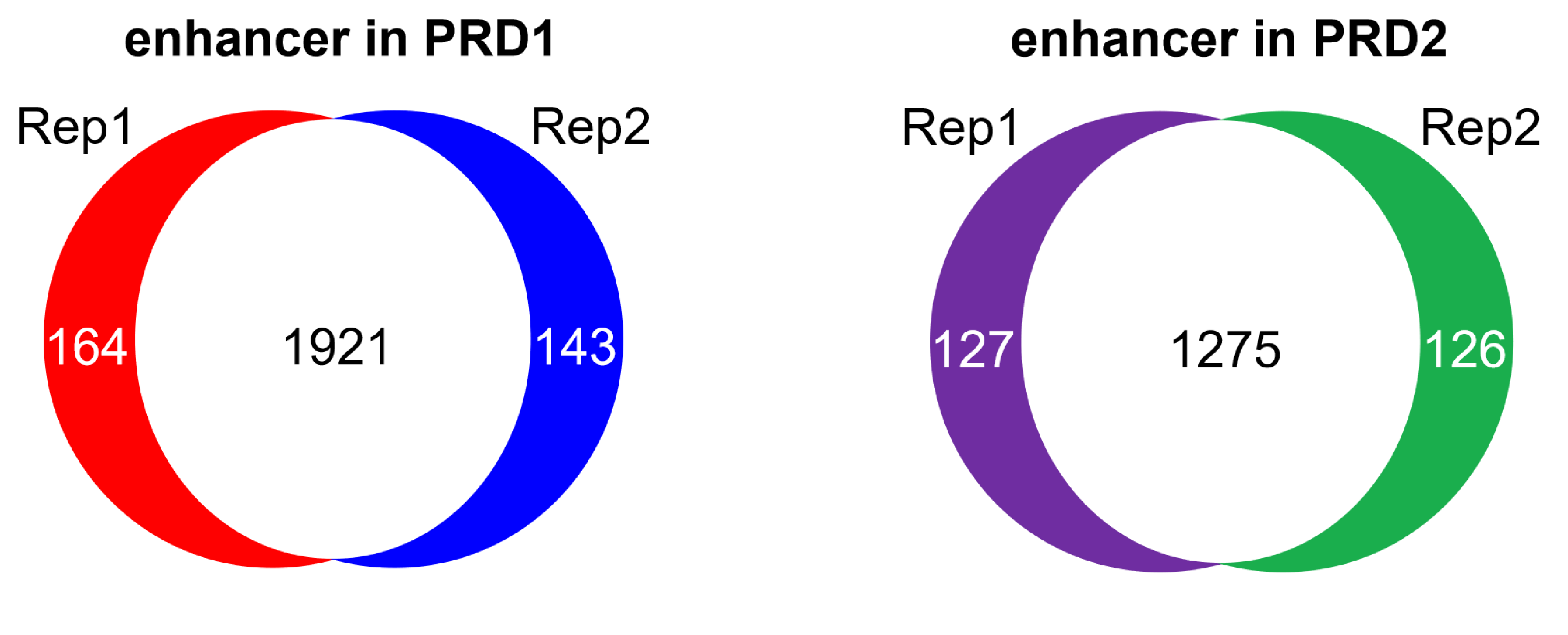

Supplement: Supplementary file 1 — Additional file 1: Figures S1–S10. Figure S1. Overview of GRO-seq library construction. A, C Isolated liver nuclei stained with DAPI. B, D Amplified DNA library range from 200–500 bp. Figure S2. Agarose gel extraction for cDNA fragment from 150nt-500nt in GRO-seq library construction. Figure S3. Impact of maternal PRD on total transcripts. Differentially expressed genes between the offspring of the dams fed the PRD and NCD at the age of 4 weeks (A) and 7 weeks (B) in RNA-seq were analyzed by Kyoto Encyclopedia of Genes and Genomes (KEGG). Fold Change > 1.5, p < 0.05. Figure S4. Promoter-proximal changed genes in PRD2. A Heatmap of log2-transformed fold changes in RNA polymerases ± 5 kb from TSSs with 200 bp bin size for genes showing significant change in RNA polymerases in promoter-proximal regions (pp up: upregulated in promoter-proximal regions; pp. down: downregulated in promoter-proximal regions; gb up: upregulated in gene body region; gb down: downregulated in gene body region; gb unchanged: unchanged in gene body region). B Promoter-proximal changed genes between the offspring of the dams fed the PRD and NCD at the age of 7 weeks were analyzed by Kyoto Encyclopedia of Genes and Genomes (KEGG). fold change > 1.5, p < 0.05. Figure S5. High confidence enhancers identification. Overlap of enhancers identified in the mouse liver GRO-seq from two independent replicates prepared from 4 and 7 weeks, respectively. Figure S6. Correlation of RNA transcriptional abundance in gene body regions associated with up- (PRD1) and down- (PRD2) regulated enhancers for the closest and other active genes. Figure S7. Examination of serum lipids profiles for 7-week-old PRD mice. Figure S8. Validation results for the change of enhancers-induced metabolic genes. A Q-PCR detection for 10 randomly selected genes in total RNA of NCD1 vs PRD1, and NCD2 vs PRD2 mice livers. B Heatmap of 10 randomly chosen eRNA expression results generated by IMAGE based on their transcription in PRD1 and PR [file 13072_2021_392_MOESM1_ESM.zip › Figure S5.tif]

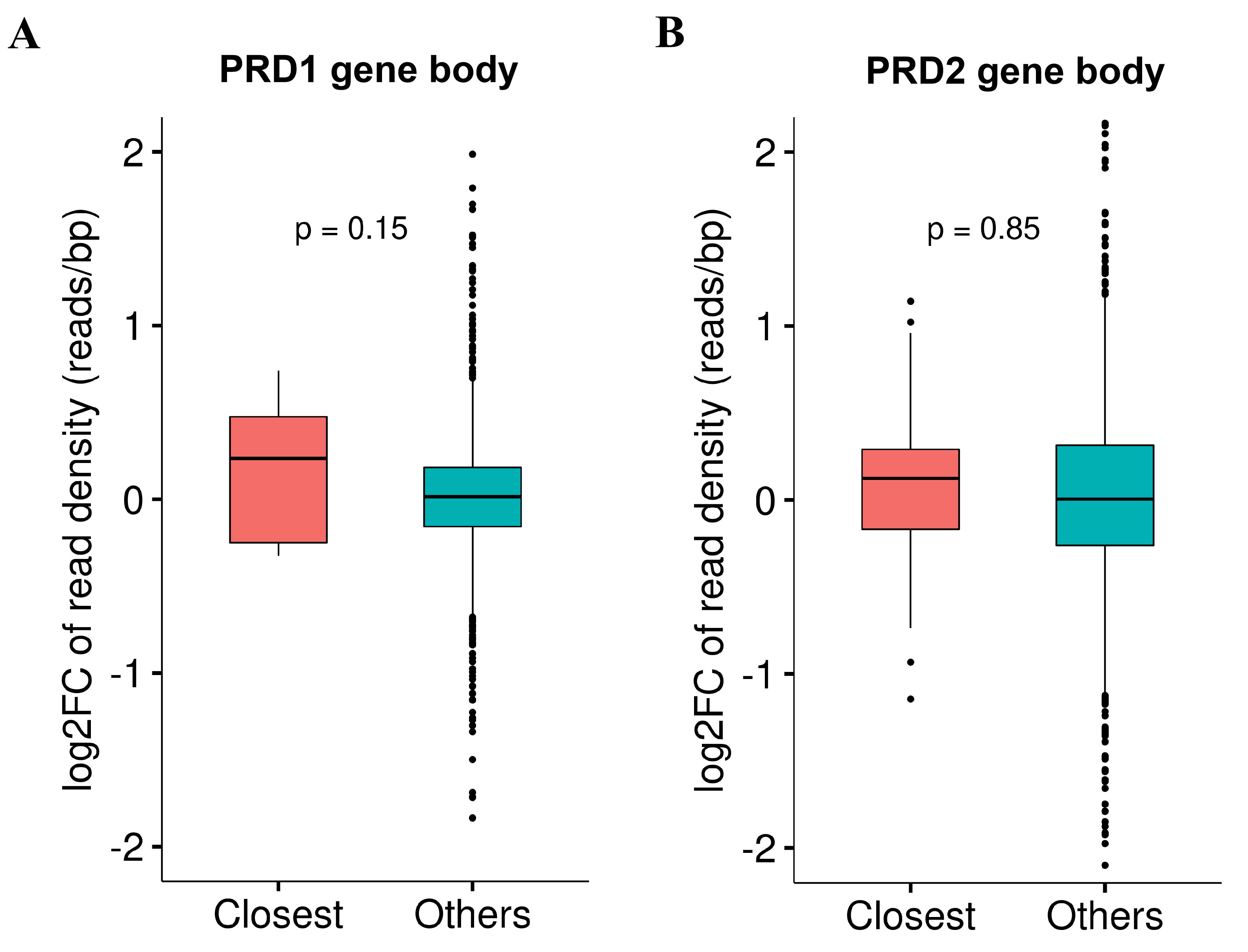

Supplement: Supplementary file 1 — Additional file 1: Figures S1–S10. Figure S1. Overview of GRO-seq library construction. A, C Isolated liver nuclei stained with DAPI. B, D Amplified DNA library range from 200–500 bp. Figure S2. Agarose gel extraction for cDNA fragment from 150nt-500nt in GRO-seq library construction. Figure S3. Impact of maternal PRD on total transcripts. Differentially expressed genes between the offspring of the dams fed the PRD and NCD at the age of 4 weeks (A) and 7 weeks (B) in RNA-seq were analyzed by Kyoto Encyclopedia of Genes and Genomes (KEGG). Fold Change > 1.5, p < 0.05. Figure S4. Promoter-proximal changed genes in PRD2. A Heatmap of log2-transformed fold changes in RNA polymerases ± 5 kb from TSSs with 200 bp bin size for genes showing significant change in RNA polymerases in promoter-proximal regions (pp up: upregulated in promoter-proximal regions; pp. down: downregulated in promoter-proximal regions; gb up: upregulated in gene body region; gb down: downregulated in gene body region; gb unchanged: unchanged in gene body region). B Promoter-proximal changed genes between the offspring of the dams fed the PRD and NCD at the age of 7 weeks were analyzed by Kyoto Encyclopedia of Genes and Genomes (KEGG). fold change > 1.5, p < 0.05. Figure S5. High confidence enhancers identification. Overlap of enhancers identified in the mouse liver GRO-seq from two independent replicates prepared from 4 and 7 weeks, respectively. Figure S6. Correlation of RNA transcriptional abundance in gene body regions associated with up- (PRD1) and down- (PRD2) regulated enhancers for the closest and other active genes. Figure S7. Examination of serum lipids profiles for 7-week-old PRD mice. Figure S8. Validation results for the change of enhancers-induced metabolic genes. A Q-PCR detection for 10 randomly selected genes in total RNA of NCD1 vs PRD1, and NCD2 vs PRD2 mice livers. B Heatmap of 10 randomly chosen eRNA expression results generated by IMAGE based on their transcription in PRD1 and PR [file 13072_2021_392_MOESM1_ESM.zip › Figure S6.tif]

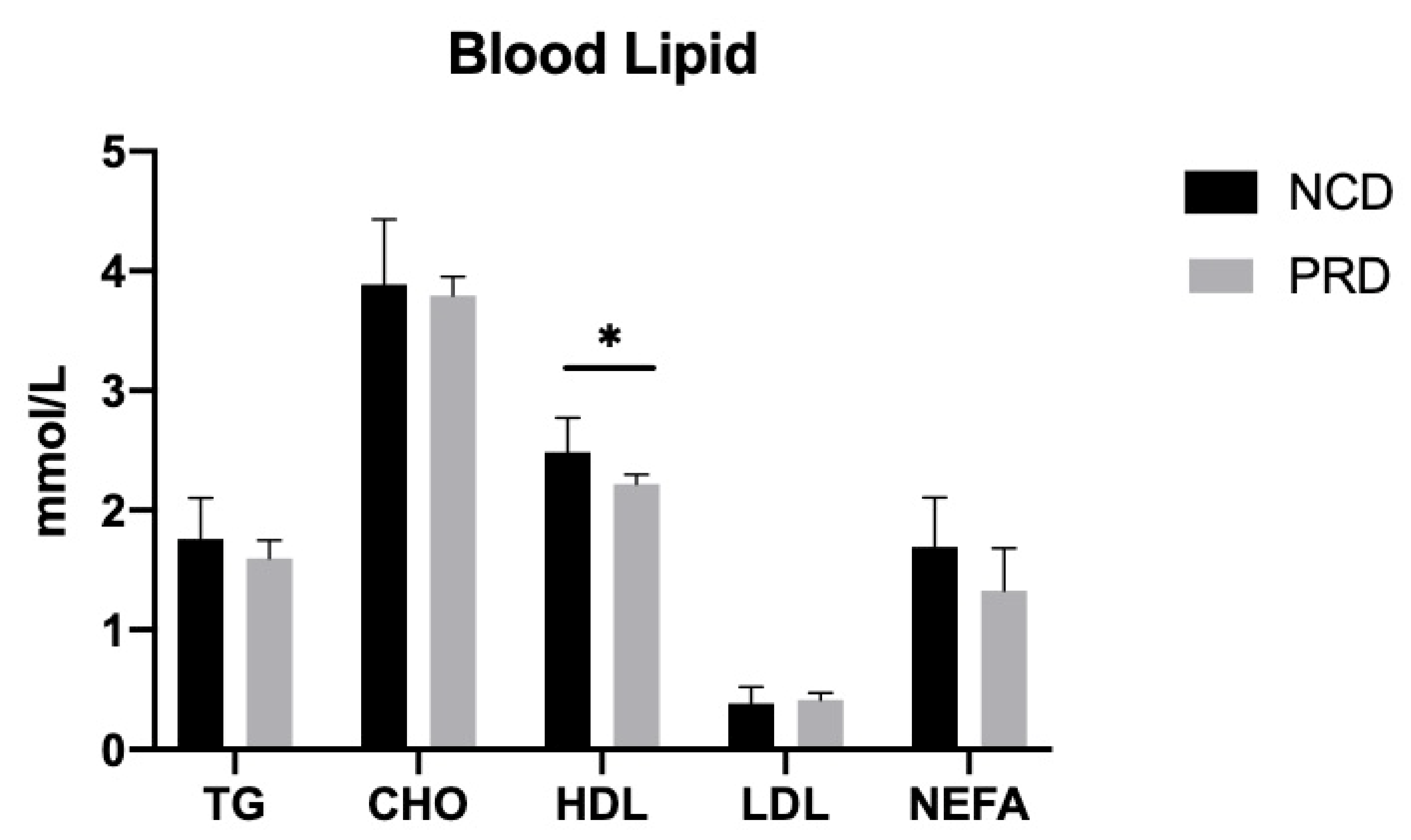

Supplement: Supplementary file 1 — Additional file 1: Figures S1–S10. Figure S1. Overview of GRO-seq library construction. A, C Isolated liver nuclei stained with DAPI. B, D Amplified DNA library range from 200–500 bp. Figure S2. Agarose gel extraction for cDNA fragment from 150nt-500nt in GRO-seq library construction. Figure S3. Impact of maternal PRD on total transcripts. Differentially expressed genes between the offspring of the dams fed the PRD and NCD at the age of 4 weeks (A) and 7 weeks (B) in RNA-seq were analyzed by Kyoto Encyclopedia of Genes and Genomes (KEGG). Fold Change > 1.5, p < 0.05. Figure S4. Promoter-proximal changed genes in PRD2. A Heatmap of log2-transformed fold changes in RNA polymerases ± 5 kb from TSSs with 200 bp bin size for genes showing significant change in RNA polymerases in promoter-proximal regions (pp up: upregulated in promoter-proximal regions; pp. down: downregulated in promoter-proximal regions; gb up: upregulated in gene body region; gb down: downregulated in gene body region; gb unchanged: unchanged in gene body region). B Promoter-proximal changed genes between the offspring of the dams fed the PRD and NCD at the age of 7 weeks were analyzed by Kyoto Encyclopedia of Genes and Genomes (KEGG). fold change > 1.5, p < 0.05. Figure S5. High confidence enhancers identification. Overlap of enhancers identified in the mouse liver GRO-seq from two independent replicates prepared from 4 and 7 weeks, respectively. Figure S6. Correlation of RNA transcriptional abundance in gene body regions associated with up- (PRD1) and down- (PRD2) regulated enhancers for the closest and other active genes. Figure S7. Examination of serum lipids profiles for 7-week-old PRD mice. Figure S8. Validation results for the change of enhancers-induced metabolic genes. A Q-PCR detection for 10 randomly selected genes in total RNA of NCD1 vs PRD1, and NCD2 vs PRD2 mice livers. B Heatmap of 10 randomly chosen eRNA expression results generated by IMAGE based on their transcription in PRD1 and PR [file 13072_2021_392_MOESM1_ESM.zip › Figure S7.tif]

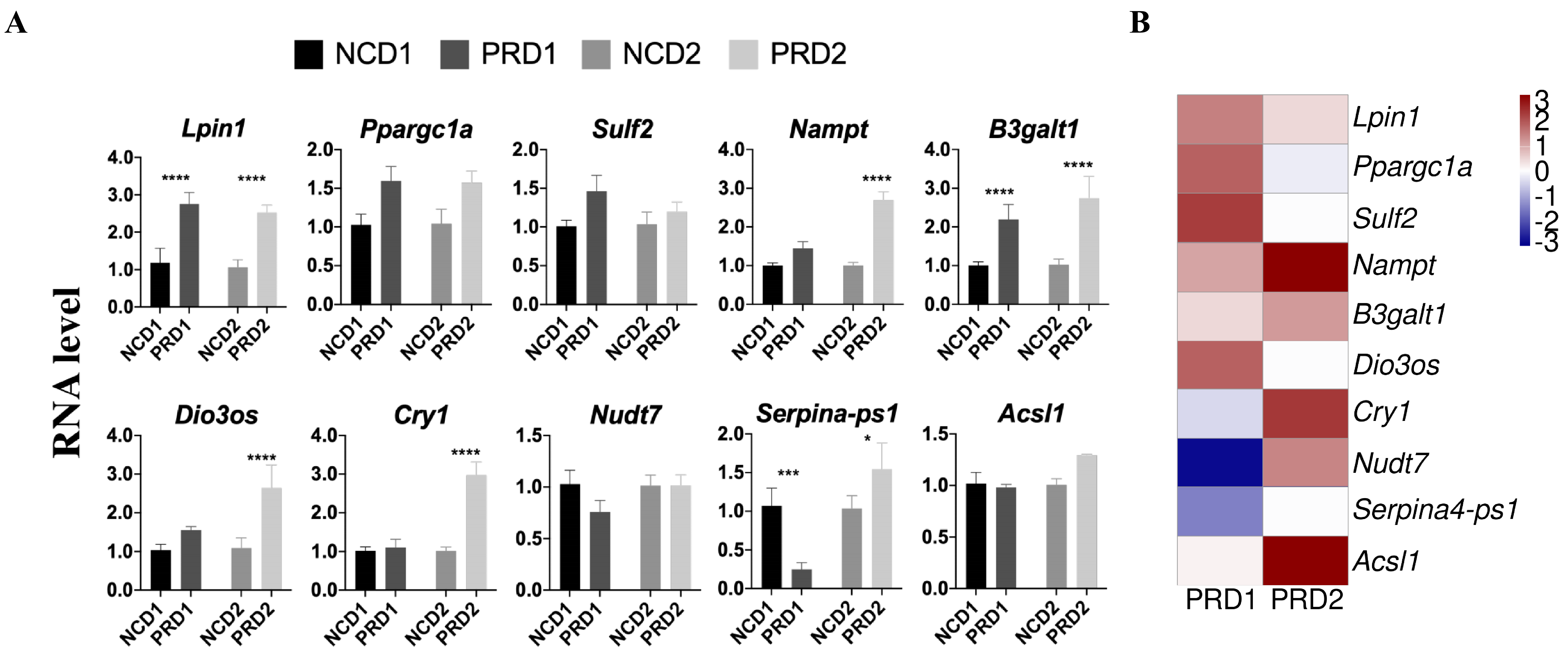

Supplement: Supplementary file 1 — Additional file 1: Figures S1–S10. Figure S1. Overview of GRO-seq library construction. A, C Isolated liver nuclei stained with DAPI. B, D Amplified DNA library range from 200–500 bp. Figure S2. Agarose gel extraction for cDNA fragment from 150nt-500nt in GRO-seq library construction. Figure S3. Impact of maternal PRD on total transcripts. Differentially expressed genes between the offspring of the dams fed the PRD and NCD at the age of 4 weeks (A) and 7 weeks (B) in RNA-seq were analyzed by Kyoto Encyclopedia of Genes and Genomes (KEGG). Fold Change > 1.5, p < 0.05. Figure S4. Promoter-proximal changed genes in PRD2. A Heatmap of log2-transformed fold changes in RNA polymerases ± 5 kb from TSSs with 200 bp bin size for genes showing significant change in RNA polymerases in promoter-proximal regions (pp up: upregulated in promoter-proximal regions; pp. down: downregulated in promoter-proximal regions; gb up: upregulated in gene body region; gb down: downregulated in gene body region; gb unchanged: unchanged in gene body region). B Promoter-proximal changed genes between the offspring of the dams fed the PRD and NCD at the age of 7 weeks were analyzed by Kyoto Encyclopedia of Genes and Genomes (KEGG). fold change > 1.5, p < 0.05. Figure S5. High confidence enhancers identification. Overlap of enhancers identified in the mouse liver GRO-seq from two independent replicates prepared from 4 and 7 weeks, respectively. Figure S6. Correlation of RNA transcriptional abundance in gene body regions associated with up- (PRD1) and down- (PRD2) regulated enhancers for the closest and other active genes. Figure S7. Examination of serum lipids profiles for 7-week-old PRD mice. Figure S8. Validation results for the change of enhancers-induced metabolic genes. A Q-PCR detection for 10 randomly selected genes in total RNA of NCD1 vs PRD1, and NCD2 vs PRD2 mice livers. B Heatmap of 10 randomly chosen eRNA expression results generated by IMAGE based on their transcription in PRD1 and PR [file 13072_2021_392_MOESM1_ESM.zip › Figure S8.tif]

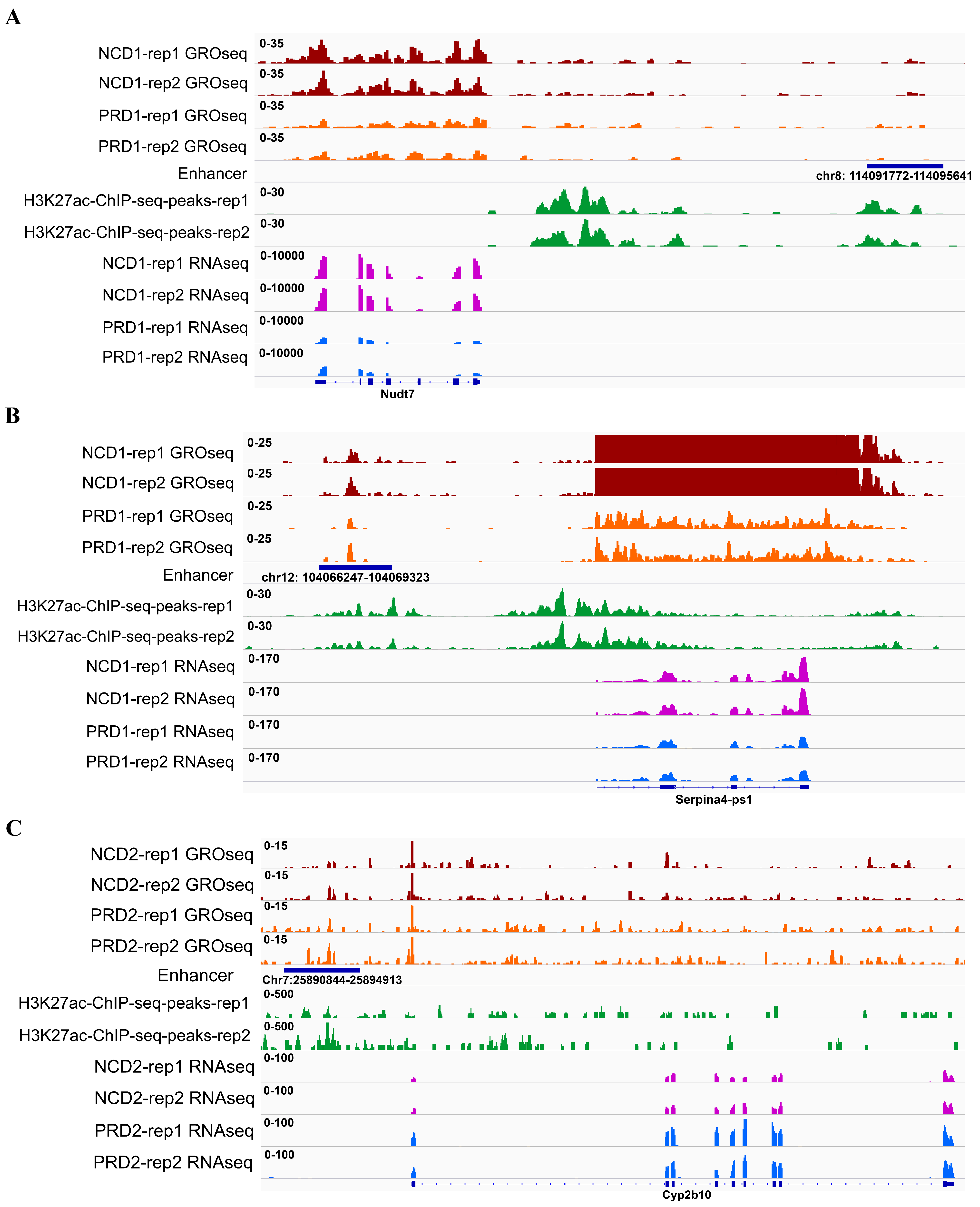

Supplement: Supplementary file 1 — Additional file 1: Figures S1–S10. Figure S1. Overview of GRO-seq library construction. A, C Isolated liver nuclei stained with DAPI. B, D Amplified DNA library range from 200–500 bp. Figure S2. Agarose gel extraction for cDNA fragment from 150nt-500nt in GRO-seq library construction. Figure S3. Impact of maternal PRD on total transcripts. Differentially expressed genes between the offspring of the dams fed the PRD and NCD at the age of 4 weeks (A) and 7 weeks (B) in RNA-seq were analyzed by Kyoto Encyclopedia of Genes and Genomes (KEGG). Fold Change > 1.5, p < 0.05. Figure S4. Promoter-proximal changed genes in PRD2. A Heatmap of log2-transformed fold changes in RNA polymerases ± 5 kb from TSSs with 200 bp bin size for genes showing significant change in RNA polymerases in promoter-proximal regions (pp up: upregulated in promoter-proximal regions; pp. down: downregulated in promoter-proximal regions; gb up: upregulated in gene body region; gb down: downregulated in gene body region; gb unchanged: unchanged in gene body region). B Promoter-proximal changed genes between the offspring of the dams fed the PRD and NCD at the age of 7 weeks were analyzed by Kyoto Encyclopedia of Genes and Genomes (KEGG). fold change > 1.5, p < 0.05. Figure S5. High confidence enhancers identification. Overlap of enhancers identified in the mouse liver GRO-seq from two independent replicates prepared from 4 and 7 weeks, respectively. Figure S6. Correlation of RNA transcriptional abundance in gene body regions associated with up- (PRD1) and down- (PRD2) regulated enhancers for the closest and other active genes. Figure S7. Examination of serum lipids profiles for 7-week-old PRD mice. Figure S8. Validation results for the change of enhancers-induced metabolic genes. A Q-PCR detection for 10 randomly selected genes in total RNA of NCD1 vs PRD1, and NCD2 vs PRD2 mice livers. B Heatmap of 10 randomly chosen eRNA expression results generated by IMAGE based on their transcription in PRD1 and PR [file 13072_2021_392_MOESM1_ESM.zip › Figure S9.tif]
