# Supplementary material for: Early-life undernutrition induces enhancer RNA remodeling in mice liver
Source: Epigenetics Chromatin. 2021 Mar 31;14:18. doi: 10.1186/s13072-021-00392-w (PMC8011416; doi:10.1186/s13072-021-00392-w)
Supplement: Supplementary file 3 — Additional file 3: Table S2–S3. Table S2. Details of the nascent transcription from GRO-seq data. Table S3. Differentially expressed (up and down regulation) eRNAs of 4 weeks (PRD1) and 7 weeks (PRD2), respectively [file 13072_2021_392_MOESM3_ESM.zip › Table S2.docx]

**Table S2. Details of the nascent transcription from GRO-seq data**

| **Item** | **Number** |
| --- | --- |
| Active genes in PRD1-GRO-seq | 9891 |
| Active genes in PRD2-GRO-seq | 9007 |
| Total identified enhancers in PRD1-GRO-seq | 4595  (known=264; novel=4331) |
| Total identified enhancers in PRD2-GRO-seq | 4599  (known=144; novel=4455) |
| Up-regulated enhancers in PRD1 sample-GRO-seq | 76  (known=1; novel=75) |
| Down-regulated enhancers in PRD1 sample-GRO-seq | 19  (known=0; novel=19) |
| Up-regulated enhancers in PRD2 sample-GRO-seq | 679  (known=22; novel=657) |
| Down-regulated enhancers in PRD2 sample-GRO-seq | 568  (known=20; novel=548) |
