# Supplementary material for: Early-life undernutrition induces enhancer RNA remodeling in mice liver
Source: Epigenetics Chromatin. 2021 Mar 31;14:18. doi: 10.1186/s13072-021-00392-w (PMC8011416; doi:10.1186/s13072-021-00392-w)
Supplement: Supplementary file 4 — Additional file 4: Table S4. Motif enrichment at sites of down/up-regulated eRNAs in livers. [file 13072_2021_392_MOESM4_ESM.docx]

| **Motif enrichment at sites of down regulated eRNAs in livers of PRD1 mice** | | |
| --- | --- | --- |
| 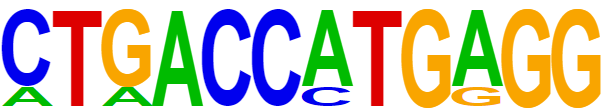 | 1e-13 | Nr5a2(NR)/Pancreas-LRH1-ChIP-Seq(GSE34295)/Homer(0.710) |
| **Motif enrichment at sites of up regulated eRNAs in livers of PRD2 mice** | | |
| Motif | p-value | Best Match |
| 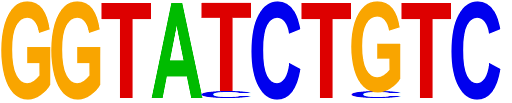 | 1e-23 | PB0036.1_Irf6_1/Jaspar(0.632) |
| 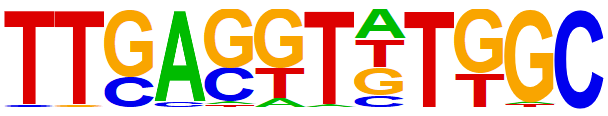 | 1e-21 | Hoxc9(Homeobox)/Ainv15-Hoxc9-ChIP-Seq(GSE21812)/Homer(0.640) |
| 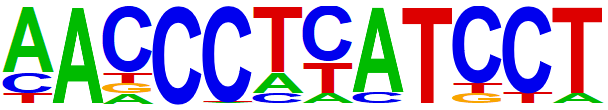 | 1e-18 | ETS:RUNX(ETS,Runt)/Jurkat-RUNX1-ChIP-Seq(GSE17954)/Homer(0.727) |
| 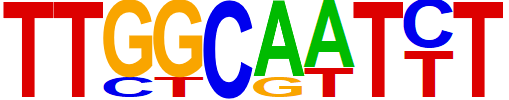 | 1e-17 | NFIA/MA0670.1/Jaspar(0.766) |
| 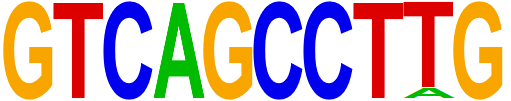 | 1e-17 | Mafb/MA0117.2/Jaspar(0.677) |
| 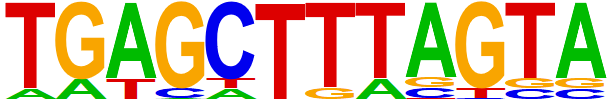 | 1e-16 | NR4A1/MA1112.1/Jaspar(0.667) |
| 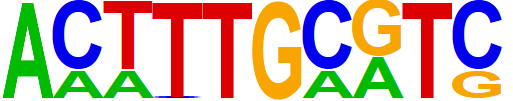 | 1e-16 | POU5F1/MA1115.1/Jaspar(0.723) |
| 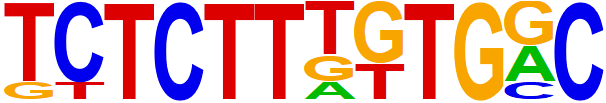 | 1e-15 | SOX10/MA0442.2/Jaspar(0.648) |
| 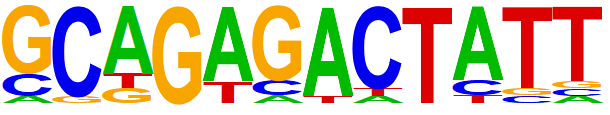 | 1e-14 | Gfi1b(Zf)/HPC7-Gfi1b-ChIP-Seq(GSE22178)/Homer(0.647) |
| 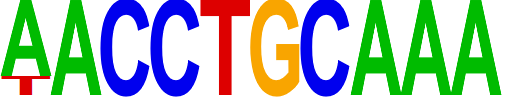 | 1e-14 | POU2F2/MA0507.1/Jaspar(0.674) |
| 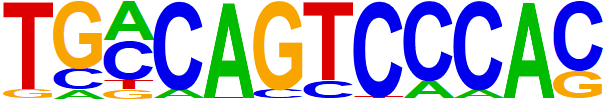 | 1e-14 | PB0114.1_Egr1_2/Jaspar(0.667) |
| 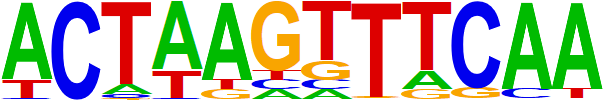 | 1e-14 | CHR/Hela-CellCycle-Expression/Homer(0.644) |
| 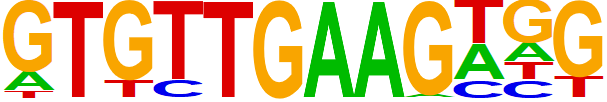 | 1e-14 | NKX2-8/MA0673.1/Jaspar(0.646) |
| 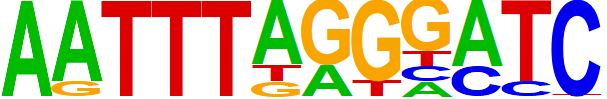 | 1e-14 | PB0162.1_Sfpi1_2/Jaspar(0.633) |
| 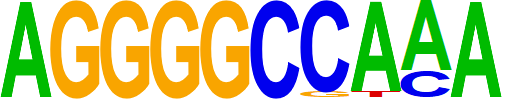 | 1e-14 | PB0118.1_Esrra_2/Jaspar(0.742) |
| 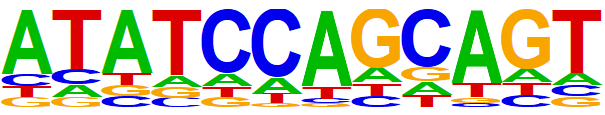 | 1e-13 | Tcf21(bHLH)/ArterySmoothMuscle-Tcf21-ChIP-Seq(GSE61369)/Homer(0.615) |
| 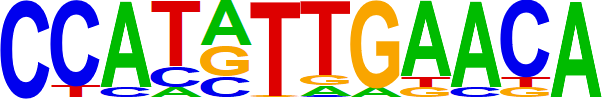 | 1e-12 | YY1/MA0095.2/Jaspar(0.676) |
| 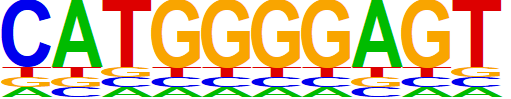 | 1e-12 | MZF1/MA0056.1/Jaspar(0.731) |
| 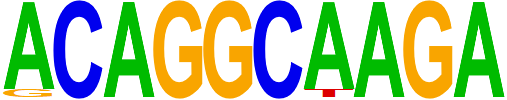 | 1e-12 | SD0002.1_at_AC_acceptor/Jaspar(0.780) |
