# Supplementary material for: Early-life undernutrition induces enhancer RNA remodeling in mice liver
Source: Epigenetics Chromatin. 2021 Mar 31;14:18. doi: 10.1186/s13072-021-00392-w (PMC8011416; doi:10.1186/s13072-021-00392-w)
Supplement: Supplementary file 6 — Additional file 6: Table S6. Details of the library primers. [file 13072_2021_392_MOESM6_ESM.docx]

**Table S3. Details of the library primers**

| **Item** | **Primer Sequence** |
| --- | --- |
| R | AATGATACGGCGACCACCGAGATCTACACTATAGCCTACACTCTTTCCCTACACGACGCTCTTCCGATC |
| NCD1-F | CAAGCAGAAGACGGCATACGAGATCGAGTAATGTGACTGGAGTTCAGACGTGTGCTCTTCCGATC |
| PRD1-F | CAAGCAGAAGACGGCATACGAGATTCTCCGGAGTGACTGGAGTTCAGACGTGTGCTCTTCCGATC |
| NCD2-F | CAAGCAGAAGACGGCATACGAGATAATGAGCGGTGACTGGAGTTCAGACGTGTGCTCTTCCGATC |
| PRD2-F | CAAGCAGAAGACGGCATACGAGATGGAATCTCGTGACTGGAGTTCAGACGTGTGCTCTTCCGATC |
