# Supplementary material for: Early-life undernutrition induces enhancer RNA remodeling in mice liver
Source: Epigenetics Chromatin. 2021 Mar 31;14:18. doi: 10.1186/s13072-021-00392-w (PMC8011416; doi:10.1186/s13072-021-00392-w)
Supplement: Supplementary file 7 — Additional file 7: Table S7. Sequences for real-time PCR primers in this study. [file 13072_2021_392_MOESM7_ESM.docx]

**Table S4. Sequences for real-time PCR primers in this study**

| Gene name | Sequences (59-39) |
| --- | --- |
| *Lpin1 F* | AAGAGACTGACAACGATCAGGA |
| *Lpin1 R* | TTCCCCAGAGAACCAGTGGAT |
| *Ppargc1a F* | TATGGAGTGACATAGAGTGTGCT |
| *Ppargc1a R* | GTCGCTACACCACTTCAATCC |
| *Sulf2 F* | CTGCCACTATGGCTGCTGTC |
| *Sulf2 R* | GTTGGGCCGGATGTTCCTG |
| *Nampt F* | GCAGAAGCCGAGTTCAACATC |
| *Nampt R* | TTTTCACGGCATTCAAAGTAGGA |
| *B3galt1 F* | GTCCGCAGTAAGTGGTATATGC |
| *B3galt1 R* | GAGCGAGGTCTTGTAAATGAGTT |
| *Dio3os F* | AGCACTCACAGGGGCCTTCTCT |
| *Dio3os R* | TCCTTCAGGTGGGAAGTGCTGA |
| *Cry1 F* | CACTGGTTCCGAAAGGGACTC |
| *Cry1 R* | CTGAAGCAAAAATCGCCACCT |
| *Nudt7 F* | AAGGCTCGCCTGAGAAAGTC |
| *Nudt7 R* | CTGAGCGGACCGTGAACAT |
| *Serpina-ps1 F* | TGGTGATATATTCCAGCAGCAC |
| *Serpina-ps1 R* | CTAGGAGGGTCTCTCCCACTTT |
| *Acsl1 F* | TGCCAGAGCTGATTGACATTC |
| *Acsl1 R* | GGCATACCAGAAGGTGGTGAG |
| *Actin F* | GGCTGTATTCCCCTCCATCG |
| *Actin R* | CCAGTTGGTAACAATGCCATGT |
| *Cry1*-eRNA-1 F | GGCACCTCACGTTTCTGAAG |
| *Cry1*-eRNA-1 R | CAGAACTATGCCTCCTCCCC |
| *Cry1*-eRNA-2 F | CACCGGCACCTCACGTTT |
| *Cry1*-eRNA-2 R | ATCCCACGCGAGAACTCAG |

*F*, Forward; *R*, Reverse
